# Dynamic capacity allocation in a radiology service considering different types of patients, individual no-show probabilities, and overbooking

**DOI:** 10.1186/s12913-021-06918-y

**Published:** 2021-09-14

**Authors:** Rodolfo Benedito Zattar da Silva, Flávio Sanson Fogliatto, André Krindges, Moiseis dos Santos Cecconello

**Affiliations:** 1 Industrial & Transportation Engineering Department, Universidade Federal do Rio Grande do Sul, Avenida Osvaldo Aranha, 99, 5° Andar, Porto Alegre, 90040-060 Brazil; 2 Mathematics Department, Universidade Federal de Mato Grosso, Cuiabá, Brazil

**Keywords:** Capacity allocation, Radiology services, Markov decision processes, No-show, Overbooking

## Abstract

**Background:**

We propose a mathematical model formulated as a finite-horizon Markov Decision Process (MDP) to allocate capacity in a radiology department that serves different types of patients. To the best of our knowledge, this is the first attempt at considering radiology resources with different capacities and individual no-show probabilities of ambulatory patients in an MDP model. To mitigate the negative impacts of no-show, overbooking rules are also investigated.

**Methods:**

The model’s main objective is to identify an optimal policy for allocating the available capacity such that waiting, overtime, and penalty costs are minimized. Optimization is carried out using traditional dynamic programming (DP). The model was applied to real data from a radiology department of a large Brazilian public hospital. The optimal policy is compared with five alternative policies, one of which resembles the one currently used by the department. We identify among alternative policies the one that performs closest to the optimal.

**Results:**

The optimal policy presented the best performance (smallest total daily cost) in the majority of analyzed scenarios (212 out of 216). Numerical analyses allowed us to recommend the use of the optimal policy for capacity allocation with a double overbooking rule and two resources available in overtime periods. An alternative policy in which outpatients are prioritized for service (rather than inpatients) displayed results closest to the optimal policy, being also recommended due to its easy implementation.

**Conclusions:**

Based on such recommendation and observing the state of the system at any given period (representing the number of patients waiting for service), radiology department managers should be able to make a decision (i.e., define number and type of patients) that should be selected for service such that the system’s cost is minimized.

**Supplementary Information:**

The online version contains supplementary material available at 10.1186/s12913-021-06918-y.

## Background

Diagnostic imaging facilities are highly specialized units that offer various types of services such as X-ray, ultrasound, computed tomography (CT), and magnetic resonance imaging (MRI) to different classes of patients. In general, depending on how these patients arrive at the facility, they are classified as inpatient (hospitalized), outpatient, or emergency [1, 2]. Imaging facilities are constrained resources in most healthcare systems [3], which rely on rapid examination to provide timely diagnosis and proper referral of patients to treatments. The gap between demand and capacity in those facilities results in increasing waiting times [4], and proper allocation of the available capacity is one of the most challenging activities for their managers [3].

Capacity allocation, also known as advanced scheduling, consists of deciding the number of patients to be admitted to the system and how the available capacity should be distributed among the different types of patients waiting for care [5]. The main objective is to find efficient ways to allocate service requests given the available capacity, maximizing either service level (measured as the number of patients served within a clinically acceptable waiting time) or service revenues [6]. Efficient capacity allocation may lead to operational, clinical, and economic gains, not only by providing patients with timely access to healthcare but also by reducing costs. However, patient scheduling is a complex activity due to its stochastic nature and to the existence of different levels of priority [6].

In radiology departments, inpatients, outpatients, and emergency patients arrive with different probabilities and levels of urgency, presenting different cost and revenue structures. Decisions on how to serve them must consider different care options, with potential impacts on healthcare quality and the use of available facilities [7]. The solution to such a complex decision problem requires the use of mathematical tools; when uncertainties are involved in the process, Markov Decision Processes (MDPs) may be used to model the dynamics of the system and find its best feasible solution [8].

Markov Decision Processes have been used to model capacity allocation problems in different healthcare settings; e.g., radiotherapy [9], surgical theater [10], multidisciplinary, multistage, and outpatient medical assistance programs [11]; multi-facility diagnostic centers [8]; and outpatient consultations [6, 12, 13]. Some published works addressed the problem of resource allocation in radiology services using the MDP structure (a summary of these studies’ main characteristics is available in Additional file 1: Table S1) [1, 2, 4, 5, 14–18].

In this article, we propose a mathematical formulation based on a finite horizon MDP for the complex problem of allocating dynamic capacity in radiology services considering multiple resources, different types of patients, no-show modeling, and overbooking. Our proposition incorporates two factors not yet addressed in previous studies listed in Table S1 (Additional file 1), namely: (i) multiple resources with different capacities, allowing to better represent the reality of several radiology departments in which modern equipment coexist with older ones; and (ii) no-show probabilities of outpatients scheduled for CT examinations modeled using a penalized logistic regression model, considering individual characteristics of patients and schedules (see Additional file 2 for more details).

This study was motivated by theoretical and practical aspects. This article presents some contributions to the state-of-the-art on capacity planning of radiology services when compared to the works listed in Table S1 (Additional file 1). First, we present mathematical formulations that allow determining possible states and feasible actions considering the capacity configuration, which may be adapted to situations of single or multiple resources. It is known that capacity configuration directly impacts the definition of states and actions since the number of resources available for service is considered a constraint. Second, advanced capacity allocation methods that consider individual no-show probabilities allow a better representation of radiology services, being adaptable to consider specific characteristics of patients from a given region. To take into account individual no-show probabilities in an MDP structure, it is necessary to use a different approach for calculating transition probabilities, which is done in this article. In addition, considering individual probabilities instead of an average no-show rate allows a more realistic approximation of the MDP return function, which directly impacts the decision of allocating capacity.

From a practical viewpoint, the study was motivated by concerns from the radiology service managers with the idleness of CT resources due to the no-show of outpatients and, consequently, with the large average waiting time for exams (78 days). With that in view, no-show prediction and the analysis of overbooking rules to mitigate its adverse consequences, as proposed in this article, can reduce waste in the use of resources, increase productivity and reduce waiting times, as reported in similar studies in other healthcare settings [19–22]. That is relevant, especially in the Brazilian case, characterized by a unified public health system with growing demand and lack of investments to expand service capacity.

Long waiting times for CT exams may also aggravate further the clinical status of patients affected by diverse health problems, e.g., delays in cancer diagnosis are associated with longer treatment times [23] and a higher mortality rate [24, 25]. Using a model as proposed here can improve the efficiency of the decision-making process in exam facilities, improving service rates and, consequently, reducing waiting times. Facilitated access to CT scans enhances the quality of health care for patients. CT scans allow for more effective planning of care, indicating the need for surgical procedures, reducing exploratory surgery, improving cancer monitoring and treatment, and guiding the treatment of common lesions (injury, stroke, heart disease), and reducing hospital stay [26].

## Methods

### Problem description, notation, and assumptions

We considered a radiology service with three resources (which are computed tomography equipment) with different patient processing capacities. The objective is to minimize the total cost of care, consisting of waiting, overtime, and penalty costs for not serving patients over a business day of service. The problem was modeled as a finite horizon MDP and solved using DP. Table 1 provides the notation used in this article.

**Table 1:** Notation

Symbol	Description
**Patient types**	
IP	Inpatient
OP	Outpatient
EP	Emergency patient
**Indices**	
*i*	*i* − th regular service period, *i* = 1, …, *N*
*k*	*k* − th overtime service period, *k* = 1, …, *K*
*j*	Patient type, *j* = IP, OP, EP
*h*	*h* − th outpatient scheduled for regular service period *i*, $h=1,\dots, {Ag}_i^{\mathrm{OP}}$
**Intervals**	
*t*_*i*_, *t*_(*N* + *k*)_	Start of *i* − th regular period and of *k* − th overtime period
*t*_*i* + 1_, *t*_(*N* + *k* + 1)_	End of *i* − th regular period and of *k* − th extra period
**State variables**	
${w}_i^j$	Number of type *j* patients waiting for service at the start of regular period *i*, for *j* = IP, OP, EP
${w}_{\left(N+k\right)}^j$	Number of type *j* patients waiting for service at the start of overtime period *N* + *k*, for *j* = IP, OP
*Z*	Space containing all possible states of *N* regular periods
*Z*_*i*_	Set of all feasible states at the start of regular period *i*, immediately before waiting patients are selected for service, such that *Z*_*i*_ ∈ *Z*
*z*_*i*_	State at the start of regular period *i*, such that ${z}_i=\left({w}_i^{\mathrm{IP}},{w}_i^{\mathrm{OP}},{w}_i^{\mathrm{EP}}\right)\in {Z}_i$, gives the number of IPs, OPs and EPs waiting to be served
|*z*_*i*_|	Sum of elements in state *z*_*i*_, $$ \left|{z}_i\right|={w}_i^{\mathrm{IP}}+{w}_i^{\mathrm{OP}}+{w}_i^{\mathrm{EP}} $$
*S*	Space containing all possible states of *K* overtime periods
*S*_*k*_	Set of all feasible states at the start of overtime period *k*, immediately before waiting patients are selected for service, such that *S*_*k*_ ∈ *S*
*s*_*k*_	State at the start of overtime period *k*, such that ${s}_k=\left({w}_{\left(N+k\right)}^{\mathrm{IP}},{w}_{\left(N+k\right)}^{\mathrm{OP}}\right)\in {S}_k$, gives the number of IPs and OPs waiting to be served
|*s*_*k*_|	Sum of elements in state *s*_*k*_, $$ \left|{s}_k\right|={w}_{\left(N+k\right)}^{\mathrm{IP}}+{w}_{\left(N+k\right)}^{\mathrm{OP}} $$
**Actions**	
*A*	Set of all possible actions in *N* regular periods
${A}_{z_i}$	Set of all feasible actions in state *z*_*i*_, such that ${A}_{z_i}\in A$
*a*_*i*_	Action taken in regular period *i*, such that ${a}_i=\left({a}_i^{\mathrm{IP}},{a}_i^{\mathrm{OP}},{a}_i^{\mathrm{EP}}\right)\in {A}_{z_i}$, represent all IPs, OPs and EPs selected for service
*B*	Set of all possible actions in *K* overtime periods
${B}_{s_k}$	Set of all feasible actions in state *s*_*k*_, such that ${B}_{s_k}\in B$
*b*_*k*_	Action taken in overtime period *k*, such that ${b}_k=\left({b}_{\left(N+k\right)}^{\mathrm{IP}},{b}_{\left(N+k\right)}^{\mathrm{OP}}\right)\in {B}_{s_k}$_,_ represent all IPs and OPs selected for service
**Model parameters**	
*N*	Total number of regular periods
*K*	Total number of overtime periods
${p}_i^j$	Arrival probability of patient type *j* during regular period *i*, for *j* = IP, OP, EP
*Ag*^OP^	Appointment schedule of OPs
${Ag}_i^{\mathrm{OP}}$	Number of OPs scheduled in regular period *i*
*C*_*i*_, *C*_(*N* + *k*)_	Service capacity in each service period, given by the number of equipment available in regular periods *i* and in overtime periods *N* + *k*
*wc*^*j*^	Individual waiting cost for patient type *j* during regular period, for *j* = IP, OP
*oc*^*j*^	Individual overtime cost of serving patient type *j*, for *j* = IP, OP
*pc*^*j*^	Individual penalty cost for not serving patient type *j*, for *j* = IP, OP
**Functions**	
*P*_*i*_	Transition probability between states *i* and *i* + 1
${P}_i^j$	Transition probability between states *i* and *i* + 1 for patients type *j*, such that *j* = IP, OP, EP
*wc*_*i*_	Total waiting cost during regular period *i*
*oc*_*k*_	Total overtime cost in period *k*
*pc*_*N* + *K* + 1_	Total penalty cost for not providing service to patients
*TC*_*N* + *K*_	Total overall cost during a finite horizon decision period (one business day), with *N* + *K* decision points
*V*_*i*_(*z*_*i*_)	Minimum expected cost for each regular period *i*
*V*_*k*_(*s*_*k*_)	Minimum expected cost for each overtime period *k*
*V*_*N* + *K* + 1_(*s*_*N* + *K* + 1_)	Minimum expected penalty cost

We assume a finite planning horizon of one business day with *N* regular service periods comprised of equally spaced time intervals, such that *t*_*i*_ and *t*_*i* + 1_ represent the start and end of the *i* − th regular period. Due to the use of overbooking rules, *K* overtime service periods are made available to serve patients not contemplated during regular periods, such that *t*_(*N* + *k*)_ and *t*_(*N* + *k* + 1)_ represent the start and end of the *k* − th overtime period. Overtime periods are also comprised of discrete and identical time intervals.

On each business day, three types of patients arrive independently in each regular period *i* (*i* = 1, …, *N*), namely: (*j* = 1) inpatients (IPs), (*j* = 2) outpatients (OPs), and (*j* = 3) emergency patients (EPs) . Demand for OP exams is known beforehand since they request service days or weeks in advance and are scheduled according to *Ag*^OP^. Each outpatient *h* displays an individual and independent probability of show (${p}_i^{{\mathrm{OP}}_h}$) or no-show $\Big(1-{p}_i^{{\mathrm{OP}}_h}$) which, in opposition to the existing literature, is determined considering the patient’s individual characteristics and schedules. All OPs that arrive for service are deemed punctual.

IP and EP service requests are random, generated by the hospital (wards or emergency department), and occurring with probabilities ${p}_i^{\mathrm{IP}}$ and ${p}_i^{\mathrm{EP}}$, respectively. It is assumed that one single service request will arrive for IPs and EPs during a regular period *i*, in accordance with previous related literature [1, 16].

Variables ${w}_i^{\mathrm{IP}},$ ${w}_i^{\mathrm{OP}}$ and ${w}_i^{\mathrm{EP}}$ give the number of IPs, OPs, and EPs waiting to be served at the start of regular period *i*; ${w}_{\left(N+k\right)}^{\mathrm{IP}}$ and ${w}_{\left(N+k\right)}^{\mathrm{OP}}$ give the number of IPs and OPs waiting to be served at the start of overtime period *N* + *k*. EPs are prioritized over IPs and OPs, i.e., if an EP enters the system at (*t*_*i* − 1_, *t*_*i*_], she must be selected immediately for service in the next period (*t*_*i*_, *t*_*i* + 1_]. That ensures EPs are not put on hold, incurring waiting costs. On the other hand, waiting costs *wc*^IP^ and *wc*^OP^ are incurred if IPs and OPs are not selected for service during regular periods; if they are not served in regular periods then overtime periods will be available, incurring overtime costs *oc*^IP^ and *oc*^OP^. In overtime periods, no IPs or OPs arrive for service. We assume that EPs arriving at *N* + 1 are served by dedicated CT equipment; thus, no more than two resources can be allocated to serve IPs and OPs in overtime periods. In addition, penalty costs *pc*^IP^ and *pc*^OP^ are incurred for IPs and OPs left unserved after the regular and overtime periods have elapsed. In line with previous studies [1, 16], it is assumed that IPs have a higher associated penalty cost due to the possibility of spending an extra day in the hospital. It is assumed that an unserved IP can arrive for service the next day, at any regular period. In opposition, unserviced OPs may request service at a future time according to a new appointment schedule. EPs that arrive between one business day of service and the next are served by a dedicated resource; therefore, the system starts each day in empty state $\Big({w}_0^{\mathrm{IP}}$ = ${w}_0^{\mathrm{OP}}$ = ${w}_0^{\mathrm{EP}}=0\Big)$.

**Fig. 1:**
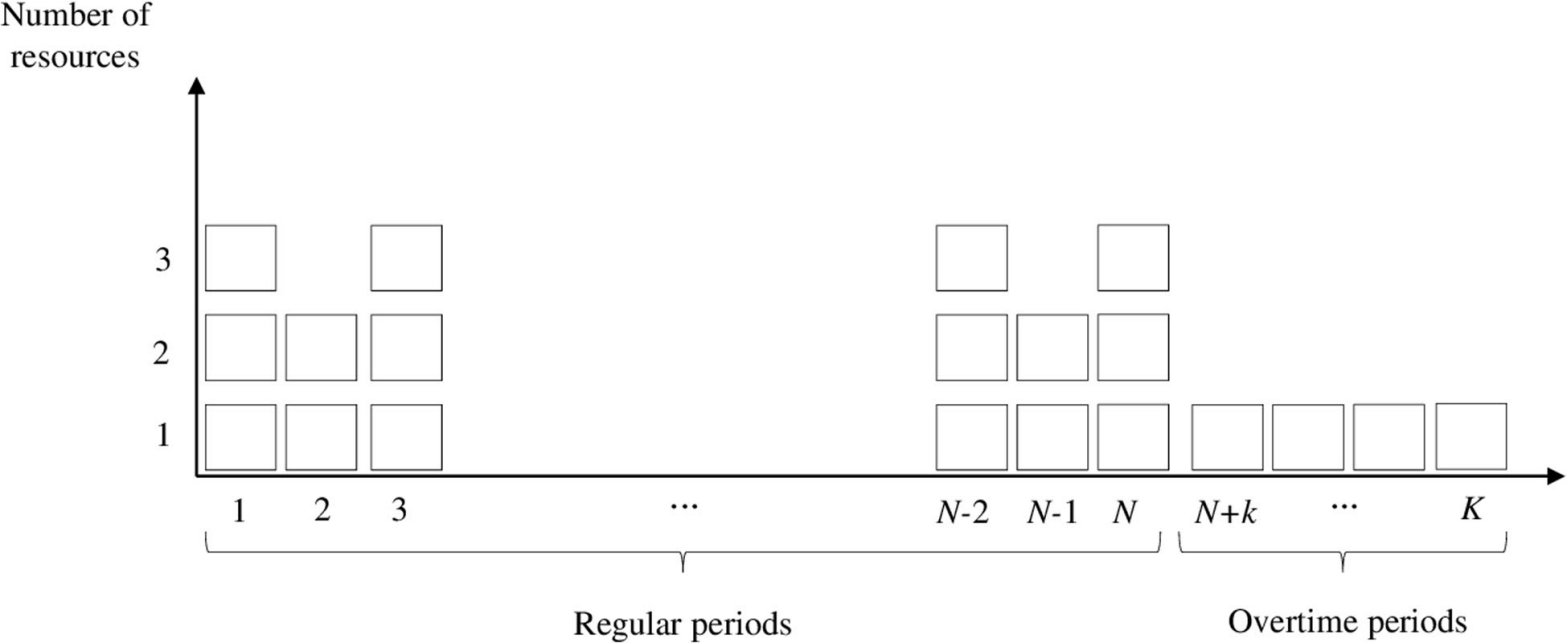
Service capacity in regular and overtime periods

The number of patients that can be served is limited by the number *C*_*i*_ and *C*_(*N* + *k*)_ of resources available in each regular and overtime period, respectively. The service time of a patient (regular or overtime) is independent of the state of the system or type of patient, being considered equal to the duration of the period. However, in the analyzed department, one of the resources has a smaller capacity (despite performing the same types of CT exams), and its associated service time is equivalent to twice that of others. In overtime periods, one or two resources can be made available to meet the demand remaining from regular periods. Service capacity in regular (considering up to 3 resources) and overtime (considering a single resource) periods is represented in Fig. 1.

### Mathematical formulation for the dynamic capacity allocation problem

The capacity allocation problem considered was modeled as a finite horizon Markov Decision Process (MDP), often used to represent systems that evolve randomly through stages through a finite number of discrete-time moments. The proposal extends the problem of dynamic resource allocation presented in Kolisch and Sickinger [1], incorporating the following factors: (i) possibility of IP and EP arrivals from the first period of regular service (*i* = 1); (ii) three resources with different service capacities; (iii) OP no-show probabilities determined based on a penalized logistic regression model considering their individual characteristics and schedules; (iv) use of overbooking to mitigate the effects of OP no-shows; and (v) use of overtime service periods to serve IPs and OPs.

### Decision stages

Assume a system that at each business day has *N* regular periods and *K* overtime periods evenly distributed in discrete time intervals. At the start of each service period (or stage), the system is observed regarding its state, and a decision (or action) is taken considering the number *C*_*i*_ and *C*_(*N* + *k*)_ of resources available.

### State space

In the proposed model, *Z* and *S* represent the sets of all possible states in *N* and *K* regular and overtime periods, respectively. Subsets *Z*_*i*_ ∈ *Z* and *S*_*k*_ ∈ *S* represent feasible states at the start of each regular period *i* and overtime period *N* + *k*, just before waiting patients of type *j* are selected to be served; *z*_*i*_ ∈ *Z*_*i*_ is a given state in *i*, such that ${z}_i=\left({w}_i^{\mathrm{IP}},{w}_i^{\mathrm{OP}},{w}_i^{\mathrm{EP}}\right)$; *s*_*k*_ ∈ *S*_*k*_ is a given state in *k*, such that ${s}_k=\left({w}_{\left(N+k\right)}^{\mathrm{IP}},{w}_{\left(N+k\right)}^{\mathrm{OP}}\right)$.

The set of all feasible states at the start of each regular period *i* is defined as follows (details are given in Additional file 3):

- For *i* = 1:

1 $$ {Z}_i=\left\{\left({w}_i^{\mathrm{IP}},{w}_i^{\mathrm{OP}},{w}_i^{\mathrm{EP}}\right)|\begin{array}{l}0\le {w}_i^{\mathrm{IP}}\le 1\\ {}0\le {w}_i^{\mathrm{OP}}\le {Ag}_i^{\mathrm{OP}}\\ {}0\le {w}_i^{\mathrm{EP}}\le 1\end{array}\right\} $$

- For *i* ≥ 2:

2 $$ {Z}_i=\left\{\begin{array}{l}\left\{\left({w}_i^{\mathrm{IP}},{w}_i^{\mathrm{OP}},{w}_i^{\mathrm{EP}}\right)|\begin{array}{l}0\le {w}_i^{\mathrm{IP}}\le i\\ {}0\le {w}_i^{\mathrm{OP}}\le \sum \limits_{l=1}^i\ {Ag}_l^{\mathrm{OP}}\\ {}0\le {w}_i^{\mathrm{EP}}\le 1\\ {}{w}_i^{\mathrm{IP}}+{w}_i^{\mathrm{OP}}\le \sum \limits_{l=1}^i{Ag}_l^{\mathrm{OP}}+i-\left[\sum \limits_{l=1}^{i-1}\left({C}_l-{w}_l^{\mathrm{EP}}\right)\right]\end{array}\right\},\\ {}\mathrm{If}\ \sum \limits_{l=1}^i\ {Ag}_l^{\mathrm{OP}}+i\ge \left[\sum \limits_{l=1}^{i-1}\left({C}_l-{w}_l^{\mathrm{EP}}\right)\right]\\ {}\left\{\left({w}_i^{\mathrm{IP}},{w}_i^{\mathrm{OP}},{w}_i^{\mathrm{EP}}\right)|\begin{array}{l}0\le {w}_i^{\mathrm{IP}}\le 1\\ {}0\le {w}_i^{\mathrm{OP}}\le {Ag}_i^{\mathrm{OP}}\\ {}0\le {w}_i^{\mathrm{EP}}\le 1\end{array}\right\},\\ {}\mathrm{otherwise}\ \end{array}\right\} $$

Note that for the analyzed system at any regular period *i* there will always be capacity available to serve IPs and/or OPs, even if one EP has been selected (since 2 ≤ *C*_*i*_ ≤ 3), meaning that the remaining capacity in *i*, given by the difference between the capacity and the number of EPs waiting $\left({C}_i-{w}_i^{\mathrm{EP}}\right)$, will be allocated to serve IPs and/or OPs.

The set of all feasible states at the start of each overtime period *N* + *k* is defined as follows (details are given in Additional file 3):

- For *k* = 1:

3 $$ {S}_k=\left\{\begin{array}{l}\left\{\left({w}_{\left(N+k\right)}^{\mathrm{IP}},{w}_{\left(N+k\right)}^{\mathrm{OP}}\right)|\begin{array}{l}{w}_{\left(N+k\right)}^{\mathrm{IP}}\le N\\ {}{w}_{\left(N+k\right)}^{\mathrm{OP}}\le \sum \limits_{l=1}^N\ {Ag}_l^{\mathrm{OP}}\\ {}{w}_{\left(N+k\right)}^{\mathrm{IP}}+{w}_{\left(N+k\right)}^{\mathrm{OP}}\le \sum \limits_{l=1}^N{Ag}_l^{\mathrm{OP}}+N-\left[\sum \limits_{l=1}^N\left({C}_l-{w}_l^{\mathrm{EP}}\right)\right]\end{array}\right\},\\ {}\mathrm{if}\ \sum \limits_{l=1}^N{Ag}_l^{\mathrm{OP}}+N\ge \left[\sum \limits_{l=1}^N\left({C}_l-{w}_l^{\mathrm{EP}}\right)\right]\\ {}\left\{\left({w}_{\left(N+k\right)}^{\mathrm{IP}},{w}_{\left(N+k\right)}^{\mathrm{OP}}\right)|\begin{array}{l}{w}_{\left(N+k\right)}^{\mathrm{IP}}=0\\ {}{w}_{\left(N+k\right)}^{\mathrm{OP}}=0\end{array}\right\},\\ {}\mathrm{otherwise}\kern0.75em \end{array}\right\} $$

- For *k* = 2, …, *K*:

4 $$ {S}_k=\left\{\left({w}_{\left(N+k\right)}^{\mathrm{IP}},{w}_{\left(N+k\right)}^{\mathrm{OP}}\right)|\begin{array}{l}{w}_{\left(N+k\right)}^{\mathrm{IP}}\le N\\ {}{w}_{\left(N+k\right)}^{\mathrm{OP}}\le \sum \limits_{l=1}^N\ {Ag}_l^{\mathrm{OP}}\\ {}\begin{array}{c}{w}_{\left(N+k\right)}^{\mathrm{IP}}+{w}_{\left(N+k\right)}^{\mathrm{OP}}\le {\sum}_{l=1}^N{Ag}_l^{\mathrm{OP}}+N-\\ {}\left[\sum \limits_{l=1}^N\left({C}_l-{w}_l^{\mathrm{EP}}\right)-\sum \limits_{y=1}^{N+k-1}{C}_{N+y}\right]\end{array}\end{array}\right\} $$

The same condition verified for eq. (2) are applicable in eqns. (3) and (4) regarding the constraint associated with the number of IPs and OPs waiting to be served; i.e. $\left({w}_{\left(N+k\right)}^{\mathrm{IP}}+{w}_{\left(N+k\right)}^{\mathrm{OP}}\right)$.

### Set of actions

In our model, *A* and *B* are the sets of all possible actions in *N* and *K* regular and overtime periods, respectively. Subsets ${A}_{z_i}\in A$ and ${B}_{s_k}\in B$ represent all feasible actions of specific system states *z*_*i*_ and *s*_*k*_, respectively. For each specific state of a regular period *i*, ${z}_i=\left({w}_i^{\mathrm{IP}},{w}_i^{\mathrm{OP}},{w}_i^{\mathrm{EP}}\right)\in {Z}_i$, a decision (or action) must be made regarding serving an EP, and the remaining capacity must be then allocated to IPs (${w}_i^{\mathrm{IP}}$) and OPs (${w}_i^{\mathrm{OP}}$) waiting to be served. Analogously, for each specific state of an overtime period *N* + *k*, ${s}_k=\left({w}_{\left(N+k\right)}^{\mathrm{IP}},{w}_{\left(N+k\right)}^{\mathrm{OP}}\right)\in {S}_k$, a decision must be made about choosing IPs (${w}_{\left(N+k\right)}^{\mathrm{IP}}$) and/or OPs (${w}_{\left(N+k\right)}^{\mathrm{OP}}$) waiting to be served, given the available capacity *C*_(*N* + *k*)_.

Decisions made in regular and overtime periods are represented by *a*_*i*_ and *b*_*k*_, respectively, such that ${a}_i=\left({a}_i^{\mathrm{IP}},{a}_i^{\mathrm{OP}},{a}_i^{\mathrm{EP}}\right)\in {A}_{z_i}$ gives the number of IPs, OPs, and EPs selected for service in regular period *i*, and ${b}_k=\left({b}_{\left(N+k\right)}^{\mathrm{IP}},{b}_{\left(N+k\right)}^{\mathrm{OP}}\right)\in {B}_{s_k}$ gives the number of IPs and OPs selected for service in overtime period *N* + *k*.

Therefore, the set ${A}_{z_i}$ of feasible actions for a given state of regular period *i* is given by:

5 $$ {A}_{z_i}=\left\{\left({a}_i^{\mathrm{IP}},{a}_i^{\mathrm{OP}},{a}_i^{\mathrm{EP}}\right)|\begin{array}{l}{a}_i^{\mathrm{IP}}\le {w}_i^{\mathrm{IP}}\\ {}{a}_i^{\mathrm{OP}}\le {w}_i^{\mathrm{OP}}\\ {}{a}_i^{\mathrm{EP}}={w}_i^{\mathrm{EP}}\\ {}{a}_i^{\mathrm{IP}}+{a}_i^{\mathrm{OP}}+{a}_i^{\mathrm{EP}}=\min \left({C}_i,\left|{z}_i\right|\right)\end{array}\right\} $$

such that $$ \left|{\mathrm{z}}_{\mathrm{i}}\right|={\mathrm{w}}_{\mathrm{i}}^{\mathrm{IP}}+{\mathrm{w}}_{\mathrm{i}}^{\mathrm{OP}}+{\mathrm{w}}_{\mathrm{i}}^{\mathrm{EP}} $$.

The set ${B}_{s_k}$ of feasible actions for a given state of overtime period *N* + *k* is given by:

6 $$ {B}_{s_k}=\left\{\left({b}_{\left(N+k\right)}^{\mathrm{IP}},{b}_{\left(N+k\right)}^{\mathrm{OP}}\right)|\begin{array}{l}{b}_{\left(N+k\right)}^{\mathrm{IP}}\le {w}_{\left(N+k\right)}^{\mathrm{IP}}\\ {}{b}_{\left(N+k\right)}^{\mathrm{OP}}\le {w}_{\left(N+k\right)}^{\mathrm{OP}}\\ {}{b}_{\left(N+k\right)}^{\mathrm{IP}}+{b}_{\left(N+k\right)}^{\mathrm{OP}}=\min \left({C}_{\left(N+k\right)},\left|{s}_k\right|\right)\end{array}\right\} $$

where $$ \left|{s}_k\right|={w}_{\left(N+k\right)}^{\mathrm{IP}}+{w}_{\left(N+k\right)}^{\mathrm{OP}} $$.

### Transition probabilities

Once decisions have been made for each regular period *i*, the random arrivals of new IPs, OPs and EPs are the only sources of uncertainty in the transition from current state (*z*_*i*_) to the next system state (*z*_*i* + 1_). Considering that in overtime periods new patients do not arrive for care, the next state of the system will depend exclusively on the decision made in the current state. Therefore, transition probabilities are only valid for determining the next states in regular periods.

As a result of choosing action ${a}_i=\left({a}_i^{\mathrm{IP}},{a}_i^{\mathrm{OP}},{a}_i^{\mathrm{EP}}\right)\in {A}_{z_i}$ in state ${z}_i=\left({w}_i^{\mathrm{IP}},{w}_i^{\mathrm{OP}},{w}_i^{\mathrm{EP}}\right)\in {Z}_i$ and considering that ${p}_i^{\mathrm{IP}}$, ${p}_i^{{\mathrm{OP}}_h}$, ${p}_i^{\mathrm{EP}}$ are the independent arrival probabilities of IPs, OPs, and EPs, the system evolves to the next state in regular period *i* + 1, denoted by ${z}_{i+1}=\left({w}_{i+1}^{\mathrm{IP}},{w}_{i+1}^{\mathrm{OP}},{w}_{i+1}^{\mathrm{EP}}\right)\in {Z}_{i+1}$, considering the following probability function:

7 $$ {P}_i\ \left.\Big({z}_{i+1}\right|{z}_i,{a}_i\left)={P}_i^{\mathrm{IP}}\ \left.\Big({w}_{i+1}^{\mathrm{IP}}\right|{w}_i^{\mathrm{IP}},{a}_i^{\mathrm{IP}}\right).{P}_i^{\mathrm{OP}}\left.\Big({w}_{i+1}^{\mathrm{OP}}\right|{w}_i^{\mathrm{OP}},{a}_i^{\mathrm{OP}}\left).{P}_i^{\mathrm{EP}}\left.\Big({w}_{i+1}^{\mathrm{EP}}\right|{w}_i^{\mathrm{EP}},{a}_i^{\mathrm{EP}}\right) $$

where *P*_*i*_ is the transition probability in time instant *t*_*i*_, ${\sum}_{z_{i+1}\in {Z}_{i+1}}{P}_i\ \left.\Big({z}_{i+1}\right|{z}_i,{a}_i\Big)=1$, ${P}_i^{\mathrm{IP}}$, ${P}_i^{\mathrm{OP}}$ and ${P}_i^{\mathrm{EP}}$ are the transition probabilities for IPs, OPs, and EPs, respectively, given by the following probability functions:

8 $$ {P}_i^{\mathrm{IP}}\ \left.\Big({w}_{i+1}^{\mathrm{IP}}\right|{w}_i^{\mathrm{IP}},{a}_i^{\mathrm{IP}}\Big)=\left\{\begin{array}{ll}1-{p}_i^{\mathrm{IP}}& \mathrm{if}\ {w}_{i+1}^{\mathrm{IP}}={w}_i^{\mathrm{IP}}-{a}_i^{\mathrm{IP}}\\ {}{p}_i^{\mathrm{IP}}& \mathrm{if}\ {w}_{i+1}^{\mathrm{IP}}={w}_i^{\mathrm{IP}}-{a}_i^{\mathrm{IP}}+1\\ {}0& \mathrm{otherwise}\end{array}\right. $$

where $\left(1-{p}_i^{\mathrm{IP}}\right)$ denotes the situation in which no new IP arrives and $\left({p}_i^{\mathrm{IP}}\right)$ denotes the arrival probability of a new IP between *i* and *i* + 1.

9 $$ {P}_i^{\mathrm{OP}}\left.\Big({w}_{i+1}^{\mathrm{OP}}\right|{w}_i^{\mathrm{OP}},{a}_i^{\mathrm{OP}}\Big)=\left\{\begin{array}{l}\left\{\sum \limits_{\beta_1+\dots +{\beta}_Q=u}\left(\prod \limits_{h=1}^Q{\left({p}_i^{{\mathrm{OP}}_h}\right)}^{\beta_h}{\left(1-{p}_i^{{\mathrm{OP}}_h}\right)}^{1-{\beta}_h}\right)\right.,\\ {}\begin{array}{c}\mathrm{if}\ {w}_{i+1}^{\mathrm{OP}}={w}_i^{\mathrm{OP}}-{a}_i^{\mathrm{OP}}+u,u=0,1,\dots, Q\ \mathrm{and}\ Q={Ag}_{i+1}^{\mathrm{OP}}\end{array}\\ {}\left\{0,\mathrm{otherwise}\right.\\ {}\ \end{array}\right. $$

such that $\sum \limits_{u=0}^Q\left(\sum \limits_{\beta_1+\dots +{\beta}_Q=u}\left(\prod \limits_{h=1}^Q{\left({p}_i^{{\mathrm{OP}}_h}\right)}^{\beta_h}{\left(1-{p}_i^{{\mathrm{OP}}_h}\right)}^{1-{\beta}_h}\right)\right)=1$, *β*_*h*_ (*h* = 1, …, *Q*) is a binary variable representing the arrivals of *u* OPs scheduled for the next regular period *i* + 1. Eq. (9) allows calculating the transition probability for OPs (${P}_i^{\mathrm{OP}}$) considering that each patient displays an independent no-show probability (1 – ${p}_i^{{\mathrm{OP}}_h}$) determined considering the patient’s characteristics and other factors (see Additional file 2).

10 $$ {P}_i^{\mathrm{EP}}\left.\Big({w}_{i+1}^{\mathrm{EP}}\right|{w}_i^{\mathrm{EP}},{a}_i^{\mathrm{EP}}\Big)=\left\{\begin{array}{ll}1-{p}_i^{\mathrm{EP}}& \mathrm{if}\ {w}_{i+1}^{\mathrm{EP}}={w}_i^{\mathrm{EP}}-{a}_i^{\mathrm{EP}}\ \mathrm{and}\ i=1,\dots, N\\ {}{p}_i^{\mathrm{EP}}& \mathrm{if}\ {w}_{i+1}^{\mathrm{EP}}={w}_i^{\mathrm{EP}}-{a}_i^{\mathrm{EP}}+1\ \mathrm{and}\ i=1,\dots, N\\ {}0& \mathrm{otherwise}\end{array}\right. $$

Interpretation of terms in Eq. (10) is simmilar to that of Eq. (8).

### Costs

Associated with each action in our model there is a cost, which may be (*i*) a waiting cost in a regular period *i*, or (*ii*) an overtime cost in an overtime period *N* + *k*. Thus, choosing an action ${a}_i=\left({a}_i^{\mathrm{IP}},{a}_i^{\mathrm{OP}},{a}_i^{\mathrm{EP}}\right)\in {A}_{z_i}$ implies in a waiting cost *wc*_*i*_(*z*_*i*_, *a*_*i*_) for IPs and OPs not selected for service, which is given by Eq. (11).

11 $$ {wc}_i\left({z}_i,{a}_i\right)={wc}^{\mathrm{IP}}.\left({w}_i^{\mathrm{IP}}-{a}_i^{\mathrm{IP}}\right)+{wc}^{\mathrm{OP}}.\left({w}_i^{\mathrm{OP}}-{a}_i^{\mathrm{OP}}\right) $$

An overtime cost *oc*_*k*_(*s*_*k*_, *b*_*k*_) is incurred whenever a given state (*s*_*k*_) of an overtime period is not the terminal state of the system, i.e., whenever ${w}_{\left(N+k\right)}^{\mathrm{IP}}>0$ and ${w}_{\left(N+k\right)}^{\mathrm{OP}}>0$. Thus, the overtime cost is also dependent on state *s*_*k*_ and chosen action *b*_*k*_, as follows:

12 $$ {oc}_k\left({s}_k,{b}_k\right)={oc}^{\mathrm{IP}}.{b}_k^{\mathrm{IP}}+{oc}^{\mathrm{OP}}.{b}_k^{\mathrm{OP}}\kern0.50em $$

A penalty cost *pc*_*N* + *K* + 1_ is incurred at period *N* + *K* + 1, being dependent on the terminal state of the system (*s*_*N* + *K* + 1_) and proportional to the number of IPs and OPs not served:

13 $$ {pc}_{N+K+1}\left({s}_{N+K+1}\right)={pc}^{\mathrm{IP}}.{w}_{\left(N+K+1\right)}^{\mathrm{IP}}+{pc}^{\mathrm{OP}}.{w}_{\left(N+K+1\right)}^{\mathrm{OP}} $$

The total cost (TC) for a finite decision-making horizon (comprised of one business day of service) with *N* + *K* decision stages is given by:

14 $$ {TC}_{N+K}=\sum \limits_{i=1}^N{wc}_i\left({z}_i,{a}_i\right)+\sum \limits_{k=1}^K{oc}_k\left({s}_k,{b}_k\right)+{pc}_{N+K+1}\left({s}_{N+K+1}\right) $$

### Value function *V* of the MDP/bellman equation

**Fig. 2:**
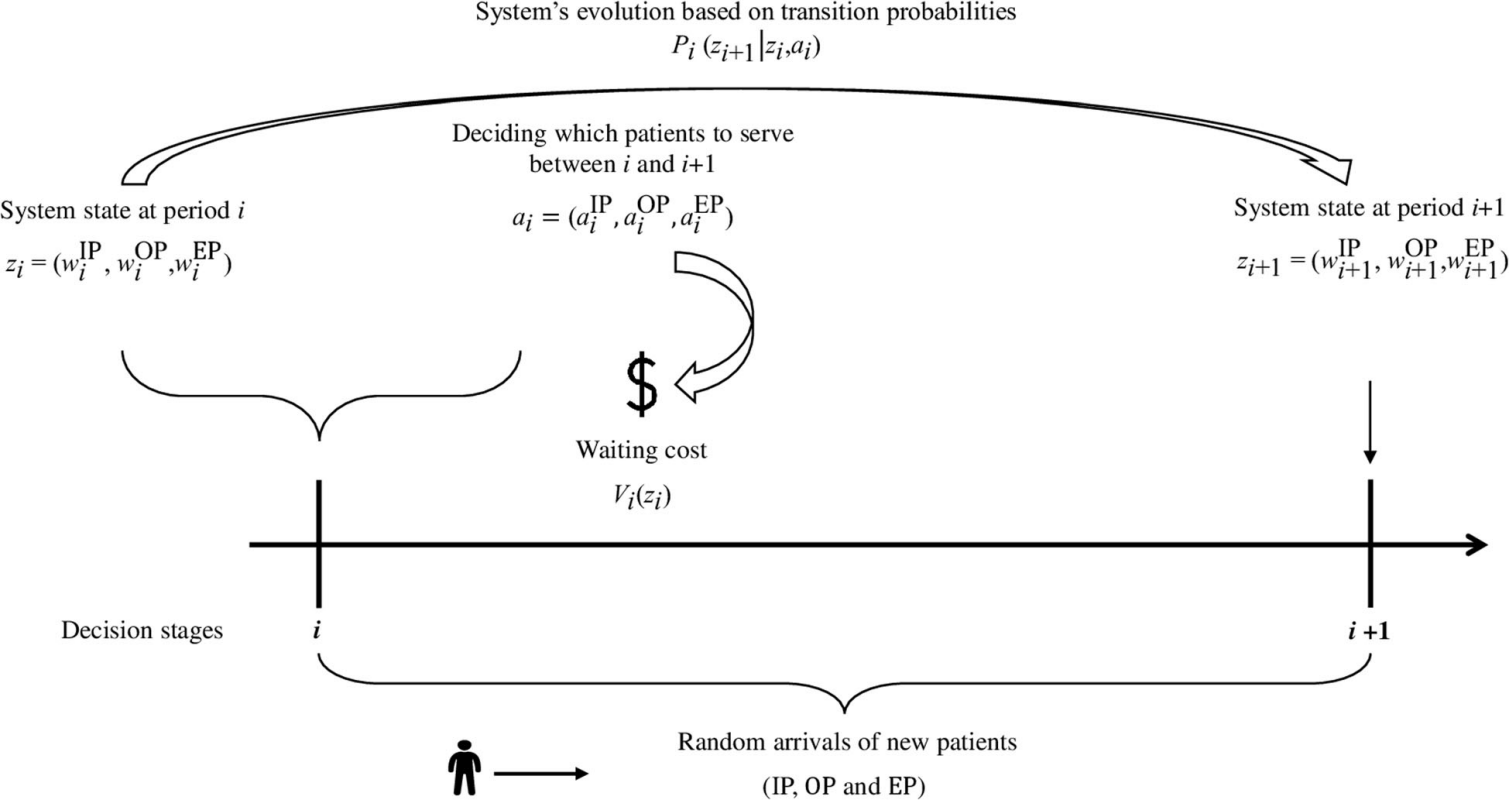
Model dynamics considering regular periods

**Fig. 3:**
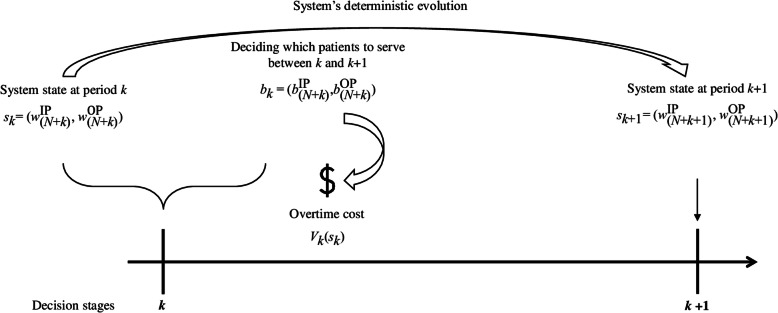
Model dynamics considering overtime periods

The aim of the model proposed here is to determine an optimal policy for allocating available capacity that defines an action for each state, such that the total cost of a business day of service is minimized. The MDP’s value function *V* represents the minimum expected cost for each state in the finite planning horizon. Considering that the model presented displays different types and cost structures for the service periods and that there are no transition probabilities in overtime periods, the minimum expected cost associated with the optimal policy is obtained by regressively and numerically solving the following recursive equations (Figs. 2 and 3 illustrate the dynamics of the proposed model in regular and overtime service periods, respectively):

- For regular periods *i* (*i* = 1, …, *N*):

15 $$ {V}_i\left({z}_i\right)=\underset{a_i\in {A}_{z_i}}{\min}\left\{{wc}_i\left({z}_i,{a}_i\right)+\sum \limits_{z_{i+1}\in {Z}_{i+1}}{P}_i\ \left.\Big({z}_{i+1}\right|{z}_i,{a}_i\Big).{V}_{i+1}\left({z}_{i+1}\right)\right\} $$

- For overtime periods *k* (*k* = 1, …, *K*):

16 $$ {V}_k\left({s}_k\right)=\underset{b_k\in {B}_{s_k}}{\min}\left\{{oc}_k\left({s}_k,{b}_k\right)+{V}_{k+1}\left({s}_{k+1}\right)\right\} $$

In addition, given that the optimal policy was used for patient selection over a business day with *N* + *K* periods, *V*_*N* + *K* + 1_ represents the expected minimum penalty cost for patients not served after regular and overtime periods (i.e., at period *N* + *K* + 1), being given by:

17 $$ {V}_{N+K+1}\left({s}_{N+K+1}\right)={pc}_{N+K+1}\left({s}_{N+K+1}\right)={pc}^{\mathrm{IP}}.{w}_{\left(N+K+1\right)}^{\mathrm{IP}}+{pc}^{\mathrm{OP}}.{w}_{\left(N+K+1\right)}^{\mathrm{OP}} $$

### Case study

We tested our propositions using real data collected from the Radiology Department of Hospital de Clinicas de Porto Alegre, a public University hospital with 850 inward beds located in southern Brazil. We focused on the computed tomography (CT) unit of that department. The work was approved by the Research Ethics Committee of the hospital under project number CAEE 83645318.6.0000.5327.

The CT unit serves IPs, OPs, and EPs, issuing approximately 30,000 reports per year. On a business day of service, between 8:00 h and 17:00 h (Monday to Friday), 74 OPs are scheduled (which corresponds to 2 OPs per regular period), in addition to 6 OPs in overbooking. Thirty-seven regular periods (*N* = 37) are available daily, in addition to 4 overtime periods (*K* = 4), all lasting 15 min. Three CT equipment are available to serve the three types of patients, two with a 15-min service time and one (older model) with a 30-min service time.

### Parameters and baseline case

The case unit serves patients from the Brazilian Unified Health System (SUS – Sistema Unico de Saude). Since the unit operates on a public budget that is made available annually, the objective function in this study is associated with costs (waiting, penalty, and overtime costs) that should be minimized not to compromise the hospital’s budget.

Waiting and penalty costs for the baseline case considered the nominal monthly per capita household income in the State in which the hospital is located [27]. In 2019, that income was US$ 343.87; considering 220 monthly hours of work, the hourly household income is US$ 1.56. The outpatient waiting cost (*wc*^OP^) was set to be US$ 1.56 in regular service periods (proportional to the patient’s potential income loss); the inpatient waiting cost (*wc*^IP^) was set as 0.5*wc*^OP^, differing from previous studies [1, 14, 16] in which *wc*^IP^ = 0. This cost is indirectly linked to longer hospital stays, making it impossible for new patients to be admitted. For each OP not served in regular and overtime periods a penalty cost *pc*^OP^ is incurred, which was set as equal to an 8-h (i.e. one workday) income loss. The penalty cost *pc*^IP^, associated with IPs, was set to be 2*pc*^OP^. The overtime cost considered the hourly wage of a radiologist in the city where the hospital is located [28], which is US$ 11.04. The overtime cost for an extra 15-min period is thus US$ 2.76.

Following Gocgun et al. [16], the baseline arrival probabilities for IPs (${p}_i^{\mathrm{IP}}$) and EPs (${p}_i^{\mathrm{EP}}$) were estimated considering the ratio between the total number of service requests for these types of patients and the total number of business days in the month of November 2019. The no-show probability of each OP was calculated using the penalized logistic regression model presented in Additional file 2. Considering individual characteristics and schedules of the 2515 patients included in the validation portion of the dataset, 100,000 random values were generated by Monte Carlo simulation for each predictor in the regression model, from which 100,000 no-show probabilities were estimated.

In the numerical analysis, we considered the baseline case and two alternative levels (low and high), as shown in Table 2.

**Table 2:** Parameter values for baseline and two alternative levels

Parameter	Patient type		
	Inpatient (IP) Baseline (low; high)	Outpatient (OP) Baseline (low; high)	Emergency (EP) Baseline (low; high)
Waiting cost (US$)	0.78 (0.39; 1.17)	1.56 (0.78; 2.34)	–
Overtime cost (US$)	2.76 (1.38; 4.14)	2.76 (1.38; 4.14)	–
Penalty cost (US$)	24.96 (12.48; 37.44)	12.48 (6.24; 18.72)	–
Arrival probability	0.60 (0.40; 0.80)	–	0.15 (0.10; 0.20)

### Overbooking rules

Two overbooking rules from the literature [17] were considered, both commonly adopted in the Brazilian Unified Health System. In the first rule, known as “double-booking” or “double” overbooking, two or more patients are scheduled for the same service period. In our study, overbooked OPs were assigned to regular service periods starting with *i* = 1 and assigning an extra patient in the subsequent sixth period (*i* + 6). Therefore, three OPs (two regular and one overbooked) were scheduled on the 1st, 7th, 13th, 19th, 25th^,^ and 31st periods. In the second rule, known as “flight” overbooking, all overbooked OPs are scheduled for the first period *i* = 1, potentially reducing resources’ idleness but increasing the waiting time for patients. In our study, eight OPs were scheduled at *i* = 1 (two regular and six overbooked).

### Alternative policies

The optimal policy obtained from solving the recursive eqns. (15) and (16) was compared with the following alternative policies, commonly reported in the literature and observed in practice:

- P1. Random selection policy: patients who wait at the start of a regular or overtime period are randomly selected for care;
- P2. Priority for OPs: OPs are prioritized over IPs. This policy is the one closest to current practice in the analyzed radiology department;
- P3. Priority for IPs: IPs have priority over OPs;
- P4. Mixed Policy 1: in the first half of regular periods, IPs are prioritized, while in the second half, the priority shifts to OPs. Since in our study *N* = 37, the first half was comprised of 19 regular periods and the second half of 18 regular periods. In case overtime periods are used, OPs are prioritized;
- P5. Mixed Policy 2: adopts a strategy opposite to that of Mixed Policy 1 in regular periods while prioritizing IPs in the case of overtime periods.

### Computational experiments

The model proposed in our study was implemented in SciLab 6.0.2, using a computer with 2.50 Giga-hertz (GHz) CPU and 8 Gigabytes (GB) RAM (the SciLab implementation code is available in Additional file 4). Two hundred sixteen different scenarios were built for comparison, based on the following configurations: 2 levels of resources (1 and 2 resources) allocated in overtime periods; 3 levels of costs (baseline, low and high); 3 arrival probabilities for IPs and EPs (baseline, low and high); 2 overbooking rules (“double” and “flight”); and 6 policies (optimal, P1, P2, P3, P4 and P5).

Optimal and alternative policies were compared in performance using the following indicators (*i*) total cost of a business day of service and (*ii*) number of IPs and OPs not served. Performance was evaluated over 10,000 business days of random events (represented by arrivals for the three types of patients) simulated for each of the 216 scenarios. Descriptive statistics (mean and standard deviation of 10,000 simulations) were computed for the two indicators considered. A *t*-test was used to compare the performance of the optimal policy and the alternative policy with the average total cost value closest to that of the optimal policy, considering a significance level of 5%.

## Results

Table 3 shows the mean and standard deviation (in parentheses) of the total cost for each policy, derived from the simulation of 10,000 days of random events. The optimal policy displayed lower average total cost when compared to alternative policies for IP and EP arrival probabilities at baseline levels (0.60 and 0.15, respectively) overall combinations of overbooking, number of resources in overtime periods, and cost levels. Policy P2 presented the average total cost closest to that of the optimal policy but different at 5% significance, reinforcing that the optimal policy should be selected to allocate the available capacity among the different types of patients.

**Table 3:** Descriptive statistics for the total cost of policies considering two types of overbooking, two levels of overtime resources, and three cost levels, with IP and EP arrival probabilities at baseline level

Type of overbooking # overtime resources Cost levels^**(2)**^	Arrival probabilities $$ {\boldsymbol{p}}_{\boldsymbol{i}}^{\mathbf{IP}}=\mathbf{0.60} $$ e $$ {\boldsymbol{p}}_{\boldsymbol{i}}^{\mathbf{EP}} $$ = 0.15					
	Optimal	P1	P2^**(1)**^	P3	P4	P5
**Double overbooking**						
**1 resource**						
Baseline level ($)	343.32 (130.78)	467.75 (171.73)	404.00* (158.27)	476.86 (172.22)	496.53 (198.91)	434.89 (148.07)
Low level ($)	169.07 (66.03)	228.85 (84.81)	198.60* (78.72)	245.11 (85.76)	254.90 (100.57)	213.18 (74.05)
High level ($)	507.79 (196.64)	702.71 (251.26)	580.32* (238.22)	720.99 (259.57)	751.90 (300.00)	637.70 (223.79)
**2 resources**						
Baseline level ($)	293.05 (120.25)	441.38 (161.73)	324.76* (146.07)	455.76 (167.32)	425.95 (198.42)	397.19 (144.94)
Low level ($)	150.58 (59.94)	215.63 (80.62)	160.48* (72.68)	228.03 (83.28)	222.88 (97.87)	194.71 (70.97)
High level ($)	441.07 (180.21)	630.40 (246.19)	489.20* (220.83)	687.53 (251.76)	631.43 (292.19)	610.11 (214.06)
**Flight overbooking**						
**1 resource**						
Baseline level ($)	410.59 (144.53)	585.43 (186.50)	467.66* (172.89)	604.80 (186.85)	613.76 (216.93)	502.70 (161.40)
Low level ($)	210.16 (72.10)	294.81 (92.41)	234.99* (86.54)	301.18 (93.32)	312.83 (106.63)	258.50 (79.98)
High level ($)	611.92 (214.32)	875.72 (276.01)	716.71* (256.26)	899.27 (277.60)	918.27 (325.10)	747.90 (240.13)
**2 resources**						
Baseline level ($)	377.21 (133.06)	551.66 (175.75)	398.56 (158.50)	571.57 (179.06)	543.33 (206.55)	479.11 (154.86)
Low level ($)	182.82 (66.20)	276.54 (88.42)	189.19 (77.43)	284.86 (90.64)	262.95 (104.33)	235.20 (76.41)
High level ($)	561.52 (197.30)	814.18 (268.57)	592.78 (234.71)	846.25 (270.12)	820.29 (314.28)	697.01 (232.77)

^(1)^Policy with total cost closest to optimal policy; ^(2)^Waiting, overtime and penalty costs set at the same level; *Value differs from the optimal policy at 5% significance level

Table 3 also shows that the “flight” overbooking rule presented the highest average total costs for all policies in all scenarios analyzed. The same behavior is observed when varying the arrival probabilities of IPs and EPs (see Tables 4 and 5), since the overbooking rule allocates a large number of regular and overtime OPs in the first service period, which consequently increases the OPs' waiting cost. When arrival probabilities of IPs and EPs are at baseline, the optimal policy with “double” overbooking and two resources is the best system configuration (with two resources in overtime periods, more patients can be served, decreasing penalty costs). The same configuration also results in lower total cost compared to the one with a single overtime resource since overtime costs are smaller than penalty costs. The same conclusion is valid for IP and EP arrival probabilities at low and high levels, as shown in Tables 4 and 5.

**Table 4:** Descriptive statistics for the total cost of policies considering two types of overbooking, two levels of overtime resources, and three cost levels, with IP and EP arrival probabilities at the low level

Type of overbooking # overtime resources Cost levels^**(2)**^	Arrival probabilities $$ {\boldsymbol{p}}_{\boldsymbol{i}}^{\mathbf{IP}}=\mathbf{0.40} $$ e $$ {\boldsymbol{p}}_{\boldsymbol{i}}^{\mathbf{EP}} $$ = 0.10					
	Optimal	P1	P2^**(1)**^	P3	P4	P5
**Double overbooking**						
**1 resource**						
Baseline level ($)	112.25 (77.45)	165.03 (110.69)	114.96* (89.18)	179.42 (115.82)	150.68 (114.73)	139.50 (94.95)
Low level ($)	55.03 (37.45)	82.52 (54.28)	55.85 (43.87)	82.68 (54.85)	73.57 (56.65)	71.09 (48.15)
High level ($)	161.53 (114.41)	222.40 (156.82)	166.57* (131.12)	258.82 (169.23)	223.99 (174.05)	212.16 (145.72)
**2 resources**						
Baseline level ($)	90.92 (63.62)	152.79 (99.96)	101.44* (62.99)	161.55 (101.63)	127.83 (91.51)	140.21 (89.69)
Low level ($)	46.75 (32.27)	80.96 (51.52)	48.60* (31.32)	82.66 (51.98)	64.37 (45.68)	66.53 (43.46)
High level ($)	143.99 (93.77)	230.29 (151.72)	146.50* (98.73)	248.54 (156.36)	196.58 (138.51)	203.99 (131.22)
**Flight overbooking**						
**1 resource**						
Baseline level ($)	169.43 (85.57)	252.00 (134.24)	170.55 (102.37)	238.96 (133.80)	240.45 (137.52)	207.82 (114.96)
Low level ($)	84.34 (44.76)	116.28 (64.76)	84.48 (50.13)	123.63 (66.59)	119.13 (68.31)	103.82 (58.20)
High level ($)	249.22 (129.76)	369.74 (199.12)	255.11* (146.80)	383.96 (203.73)	357.37 (205.57)	302.48 (171.79)
**2 resources**						
Baseline level ($)	153.10 (74.07)	240.35 (126.87)	156.41* (79.62)	246.59 (128.17)	216.77 (113.97)	206.45 (107.87)
Low level ($)	78.23 (37.65)	117.70 (61.94)	78.23 (39.90)	118.99 (63.56)	111.28 (57.93)	99.76 (52.41)
High level ($)	233.99 (114.59)	349.00 (187.17)	240.04* (119.92)	369.78 (190.75)	325.58 (170.51)	313.09 (164.14)

^(1)^Policy with total cost closest to optimal policy; ^(2)^Waiting, overtime, and penalty costs set at the same level; *Value differs from the optimal policy at 5% significance level

**Table 5:** Descriptive statistics for the total cost of policies considering two types of overbooking, two levels of overtime resources, and three cost levels, with IP and EP arrival probabilities at the high level

Type of overbooking # overtime resources Cost levels^**(3)**^	Arrival probabilities $$ {\boldsymbol{p}}_{\boldsymbol{i}}^{\mathbf{IP}}=\mathbf{0.80} $$ e $$ {\boldsymbol{p}}_{\boldsymbol{i}}^{\mathbf{EP}} $$ = 0.20					
	Optimal	P1	P2^**(1)**^	P3	P4	P5^**(2)**^
**Double overbooking**						
**1 resource**						
Baseline level ($)	656.56 (141.34)	832.58 (170.72)	758.82 (161.60)	866.45 (171.92)	923.30 (189.28)	754.50* (141.25)
Low level ($)	324.96 (70.70)	407.52 (85.61)	378.27 (81.18)	432.05 (86.26)	466.04 (92.80)	371.85* (71.10)
High level ($)	969.74 (212.71)	1258.75 (258.59)	1126.33 (242.66)	1319.41 (257.27)	1387.52 (283.22)	1118.28* (213.98)
**2 resources**						
Baseline level ($)	586.69 (140.54)	783.24 (171.00)	668.53* (161.44)	815.19 (174.62)	868.99 (205.47)	716.37 (141.86)
Low level ($)	292.47 (70.08)	398.07 (84.96)	344.99* (80.13)	418.19 (86.15)	434.20 (101.38)	353.85 (72.03)
High level ($)	882.31 (208.00)	1195.90 (254.48)	1015.75* (239.29)	1261.63 (258.62)	1282.50 (307.69)	1071.53 (214.38)
**Flight overbooking**						
**1 resource**						
Baseline level ($)	738.81 (152.76)	954.45 (176.59)	851.64 (169.44)	996.46 (177.74)	1058.32 (190.40)	820.62* (151.04)
Low level ($)	372.66 (76.82)	483.94 (87.03)	421.22 (84.62)	493.10 (88.76)	534.36 (94.74)	416.52* (75.68)
High level ($)	1116.08 (223.58)	1432.55 (270.45)	1261.66 (255.89)	1494.57 (265.11)	1595.45 (285.36)	1258.85* (224.34)
**2 resources**						
Baseline level ($)	663.58 (151.39)	927.70 (174.66)	751.88* (168.71)	953.99 (180.40)	1006.81 (212.10)	818.11 (147.15)
Low level ($)	339.78 (74.50)	464.97 (88.97)	381.60* (85.50)	482.96 (89.88)	498.59 (105.10)	400.68 (74.42)
High level ($)	993.97 (220.90)	1378.43 (262.20)	1143.96* (254.86)	1442.79 (269.03)	1507.36 (314.48)	1213.41 (226.83)

^(1),(2)^ Policy with total cost closest to optimal policy; ^(3)^Waiting, overtime, and penalty costs set at the same level; *Value differs from the optimal policy at 5% significance level

Table 4 presents the results considering IP and EP arrival probabilities at the low level (0.40 and 0.10, respectively). The optimal policy again displays the lowest average total cost, followed by policy P2; however, in some scenarios, the difference between the average total costs of the two policies was not significant since the criterion for patient selection in P2 (OPs are prioritized) is close to that of the optimal policy when IP and EP arrival probabilities are at the low level.

Table 5 presents the results considering IP and EP arrival probabilities at the high level (0.80 and 0.20, respectively). Again, the optimal policy displayed the lowest average total costs compared to alternative policies. P5 was the policy with average total costs closest to those of the optimal policy considering the two overbooking rules and 1 resource allocated in overtime periods; in the case of 2 resources, P2 was the policy closest to the optimal policy. However, both alternative policies displayed average total costs significantly different from that of the optimal policy.

**Fig. 4:**
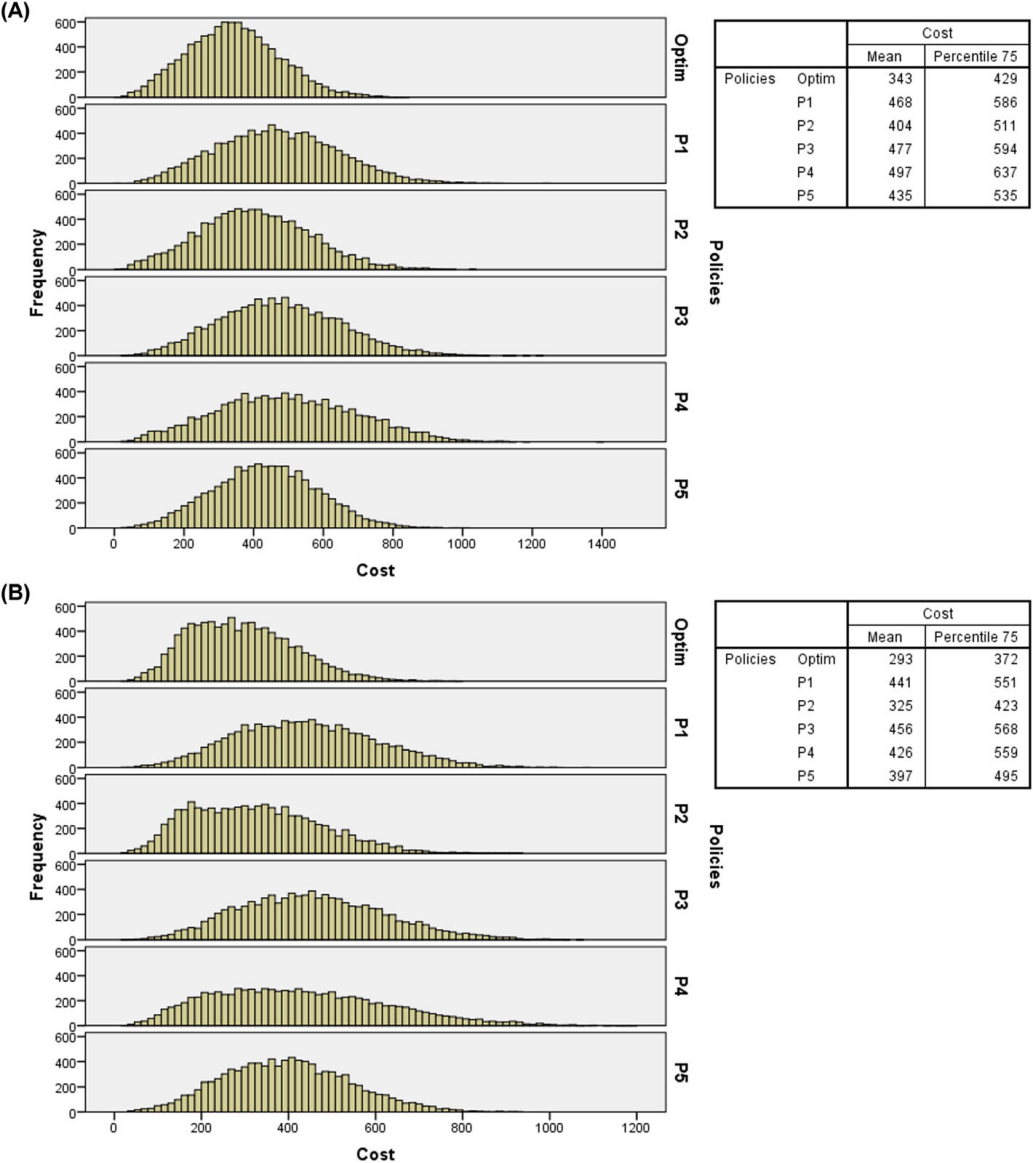
(**A**) Histograms of total costs of 10,000 days of service simulated with double overbooking, 1 (one) overtime resource and costs at baseline level (probabilities of IPs and EPs – baseline level); (**B**) Histograms of total costs of 10,000 days of service simulated with double overbooking, 2 (two) overtime resources and costs at baseline level probabilities of IPs and EPs – baseline level)

Tables 3, 4, and 5 indicate the optimal policy superior performance compared to intuitive policies, further supported by the analysis of the total costs frequency distributions for the 10,000 days of simulated random events. Figure 4 presents the histograms of arrival probabilities of IPs and EPs at baseline, considering double overbooking, two resources in overtime periods, and costs at the baseline level. The average total costs and the 75th percentile values for each policy are also presented, which indicate that 75% of the simulated days display total costs lower than these values. For example, in Fig. 4 (A), it can be seen that 75% of the 10,000 simulated days displayed costs lower than $429 for the optimal policy and $586, $511, $594, $637, and $535 for P1, P2, P3, P4, and P5, respectively.

**Fig. 5:**
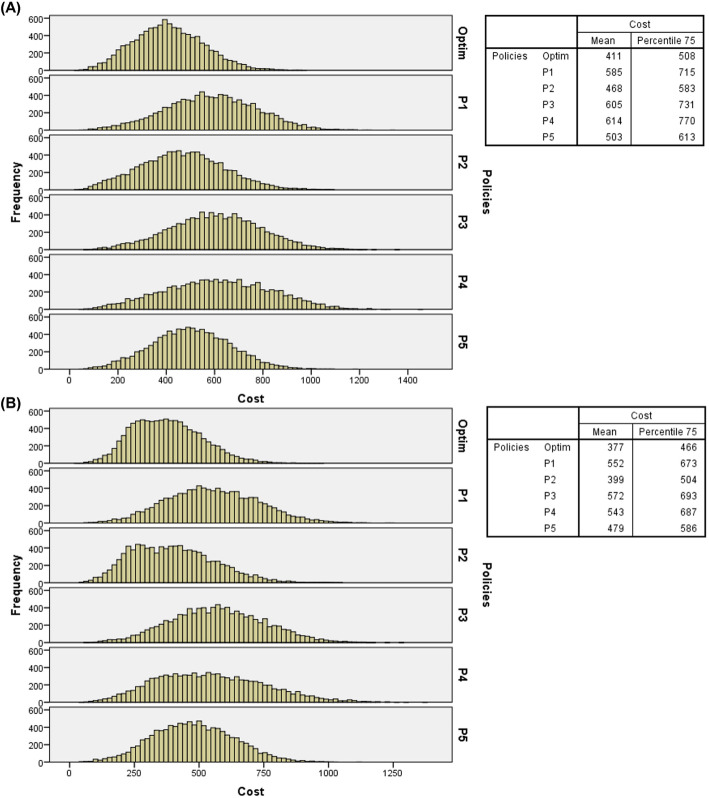
(**A**) Histograms of total costs of 10,000 days of service simulated with flight overbooking, 1 (one) overtime resource and costs at baseline level (probabilities of IPs and EPs – baseline level); (**B**) Histograms of total costs of 10,000 days of service simulated with “flight” overbooking, 2 (two) overtime resources and costs at baseline level (probabilities of IPs and EPs – baseline level)

Figure 5 also presents histograms of arrival probabilities of IPs and EPs at baseline levels, two resources in overtime periods, and costs at the baseline level, but under the “flight” overbooking rule.

**Fig. 6:**
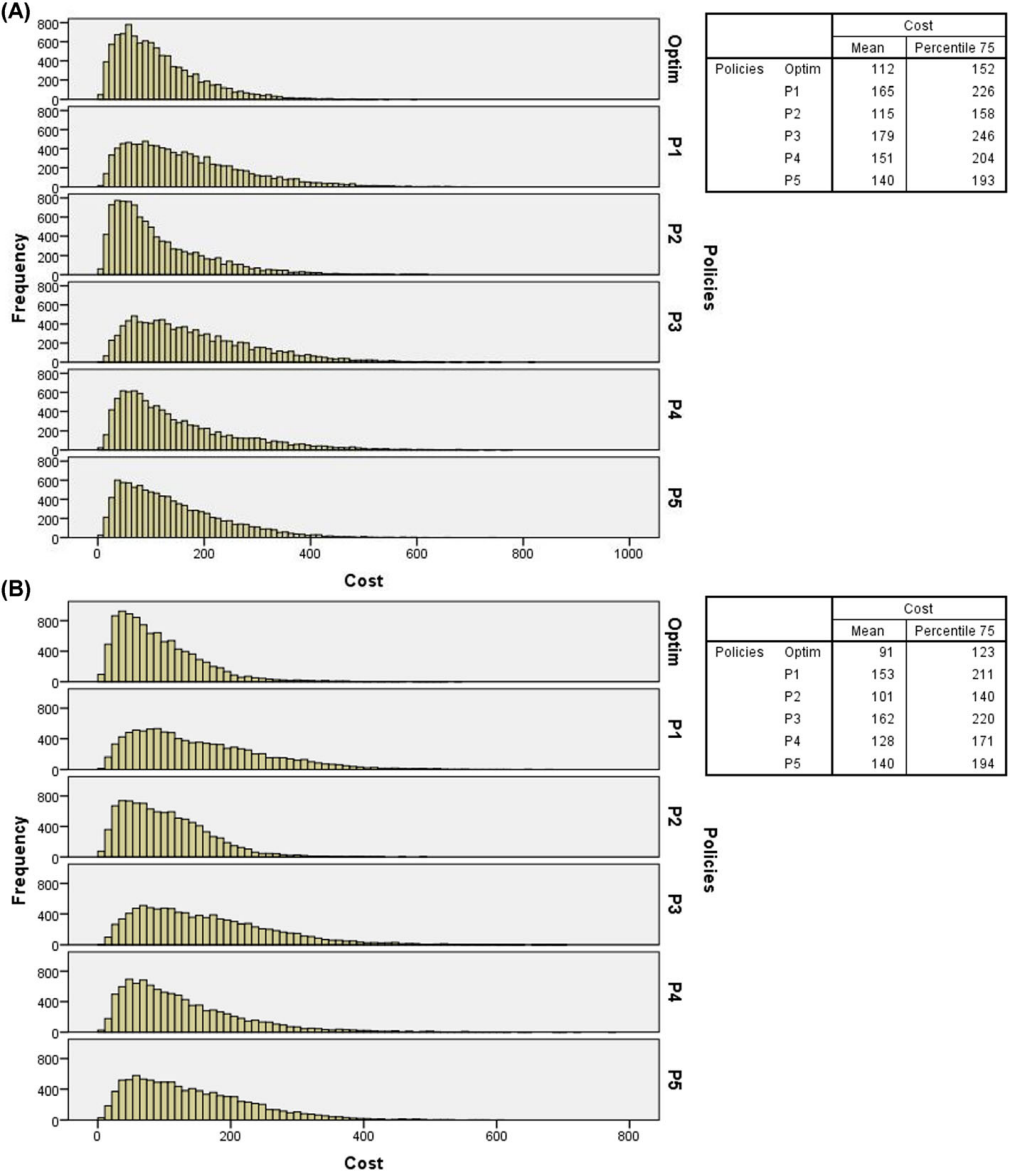
(**A**) Histograms of total costs of 10,000 days of service simulated with double overbooking, 1 (one) overtime resource and costs at baseline level (probabilities of IPs and EPs – low levels); (**B**) Histograms of total costs of 10,000 days of service simulated with double overbooking, 2 (two) overtime resources and costs at baseline level (probabilities of IPs and EPs – low level)

**Fig. 7:**
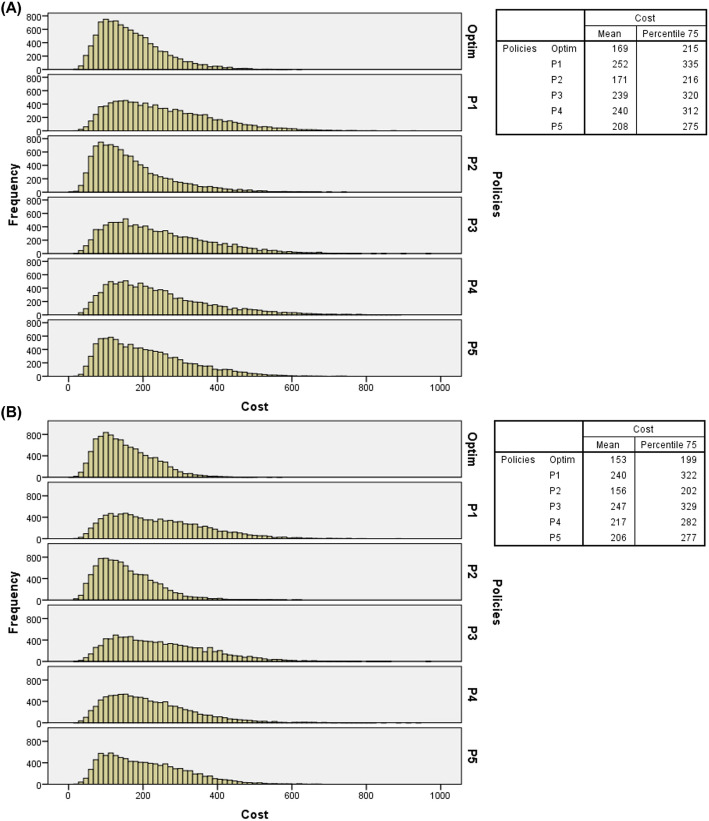
(**A**) Histograms of total costs of 10,000 days of service simulated with flight overbooking, 1 (one) overtime resource and costs at baseline level (probabilities of IPs and EPs – low levels); (**B**) Histograms of total costs of 10,000 days of service simulated with “flight” overbooking, 2 (two) overtime resources and costs at baseline level (probabilities of IPs and EPs – low level)

Figure 6 presents histograms of arrival probabilities of IPs and EPs at baseline levels, double overbooking, two resources in overtime periods, and costs at the baseline level. Figure 7 presents the resulting histograms considering the same parameters as Fig. 6 but under the “flight” overbooking rule.

**Fig. 8:**
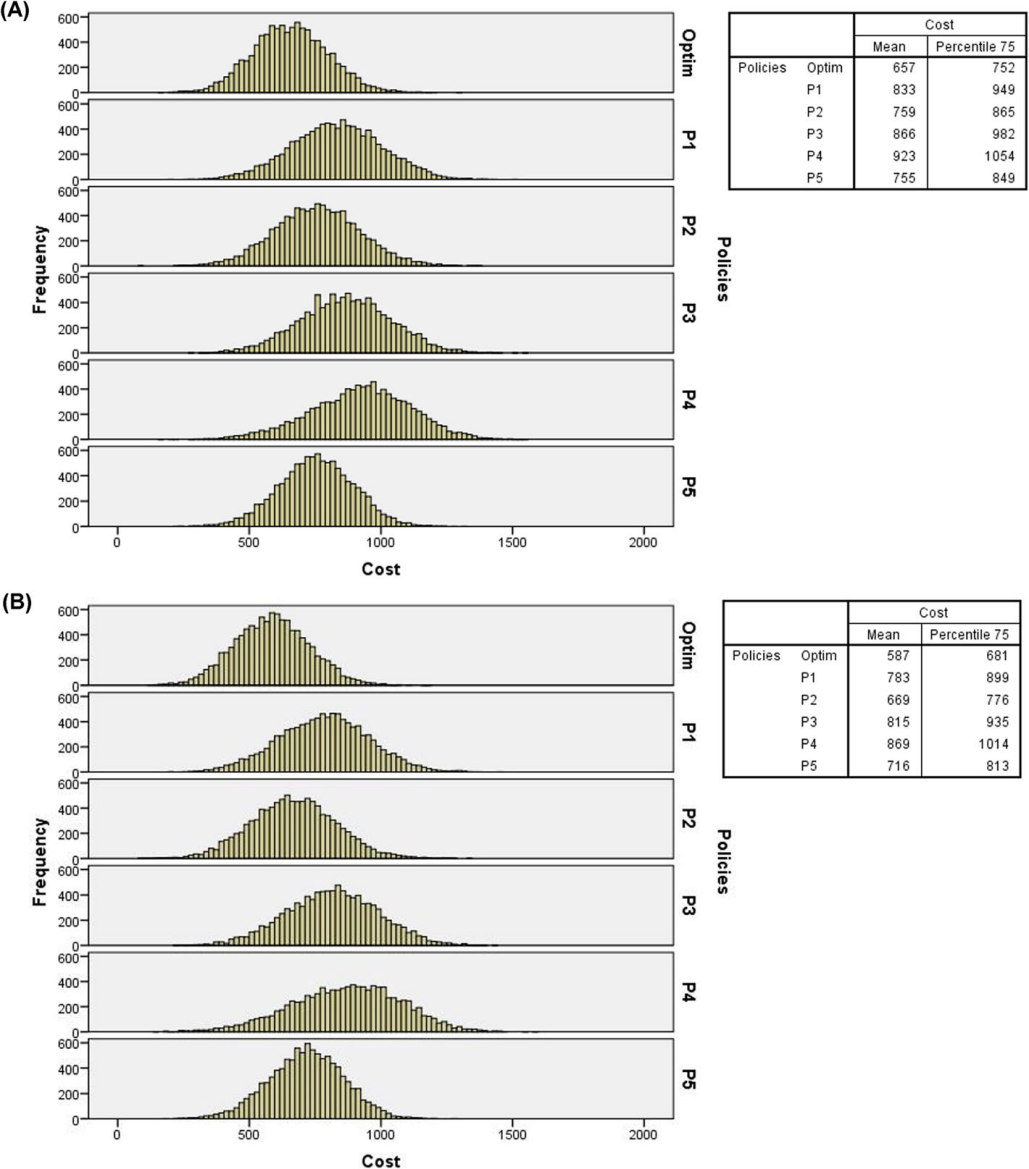
(**A**) Histograms of total costs of 10,000 days of service simulated with double overbooking, 1 (one) overtime resource and costs at baseline level (probabilities of IPs and EPs – high levels); (**B**) Histograms of total costs of 10,000 days of service simulated with double overbooking, 2 (two) overtime resources and costs at baseline level (probabilities of IPs and EPs – high level)

**Fig. 9:**
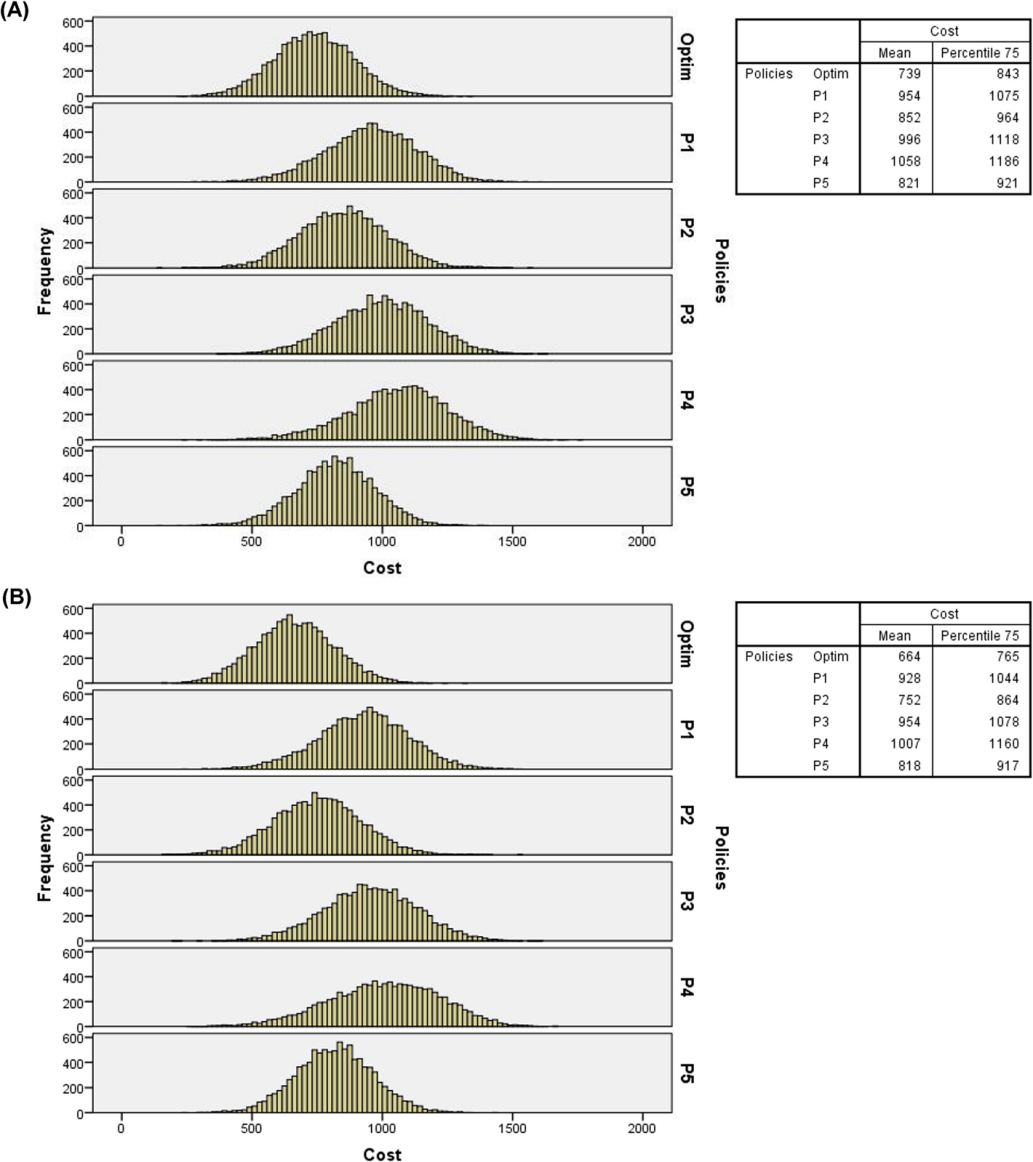
(**A**) Histograms of total costs of 10,000 days of service simulated with flight overbooking, 1 (one) overtime resource and costs at baseline level (probabilities of IPs and EPs – high levels); (**B**) Histograms of total costs of 10,000 days of service simulated with “flight” overbooking, 2 (two) overtime resources and costs at baseline level (probabilities of IPs and EPs – high level)

Figure 8 presents histograms of arrival probabilities of IPs and EPs at the high level, double overbooking, two resources in overtime periods, and costs at the baseline level. Figure 9 presents the resulting histograms considering the same parameters as Fig. 8 but under the “flight” overbooking rule.

From the frequency distributions, it can be observed that regardless of the overbooking rule, number of resources in overtime periods, levels of costs and probabilities of arrival of IPs and EPs, the optimal policy presented the lowest 75th percentile values and the lower average total costs, which further reinforces its recommended use in radiology service analyzed, particularly with IPs and EPs arrival probabilities at baseline levels (see Figs. 4 and 5) and at high levels (see Figs. 8 and 9). Additional files 5, 6, and 7, respectively, present the histograms for the three levels of IPs and EPs arrival probabilities (baseline, low and high), double and “flight” overbooking rules, 1 (one) and 2 (two) overtime resources considering the other two cost levels (low and high).

Tables 6, 7, and 8 present the mean and standard deviation values of the number of IPs and OPs not served calculated over 10,000 days of simulated random events for the 216 analyzed scenarios. The two overbooking rules resulted similar in terms of number of resources and cost levels for different arrival probability levels, reinforcing the hypothesis that the “flight” rule displays higher average total costs due to OP waiting costs.

**Table 6:** Number of IPs and OPs not served under each policy considering different scenarios, with IP and EP arrival probabilities at baseline level

	Type	Policies					
		Optimal	P1	P2	P3	P4	P5
**Double overbooking**							
**1 overtime resource**							
Costs: baseline	IP	1.79 ± 1.37	0.11 ± 0.47	7.70 ± 3.89	–	7.22 ± 3.45	–
	OP	5.68 ± 2.79	7.38 ± 3.86	–	7.44 ± 3.89	0.28 ± 0.91	7.77 ± 3.87
Costs: low	IP	1.74 ± 1.37	0.14 ± 0.52	7.43 ± 3.86	–	7.39 ± 3.45	–
	OP	5.61 ± 2.82	7.22 ± 3.82	–	7.68 ± 3.88	0.31 ± 0.95	7.61 ± 3.87
Costs: high	IP	1.81 ± 1.40	0.14 ± 0.53	7.21 ± 3.90	–	7.31 ± 3.43	–
	OP	5.77 ± 2.77	7.46 ± 3.77	–	7.39 ± 3.87	0.31 ± 0.96	7.48 ± 3.88
**2 overtime resources**							
Costs: baseline	IP	0.83 ± 1.05	0.03 ± 0.28	3.93 ± 3.37	–	3.85 ± 3.35	–
	OP	2.93 ± 2.48	3.95 ± 3.32	–	3.91 ± 3.38	0.02 ± 0.25	3.94 ± 3.38
Costs: low	IP	0.85 ± 1.05	0.02 ± 0.17	3.87 ± 3.34	–	4.13 ± 3.35	–
	OP	3.10 ± 2.50	3.74 ± 3.31	–	3.96 ± 3.35	0.02 ± 0.20	3.73 ± 3.31
Costs: high	IP	0.87 ± 1.09	0.01 ± 0.18	3.98 ± 3.38	–	3.74 ± 3.29	–
	OP	3.13 ± 2.49	3.66 ± 3.31	–	3.95 ± 3.38	0.02 ± 0.22	4.16 ± 3.41
**Flight overbooking**							
**1 overtime resource**							
Costs: baseline	IP	1.75 ± 1.40	0.19 ± 0.55	7.14 ± 3.99	–	7.01 ± 3.61	–
	OP	5.38 ± 2.86	7.31 ± 3.99	–	7.42 ± 3.97	0.28 ± 0.91	7.05 ± 3.99
Costs: low	IP	1.79 ± 1.43	0.16 ± 0.49	7.23 ± 3.99	–	7.12 ± 3.56	–
	OP	5.54 ± 2.85	7.26 ± 3.94	–	7.30 ± 3.96	0.28 ± 0.90	7.52 ± 3.96
Costs: high	IP	1.74 ± 1.41	0.24 ± 0.60	7.25 ± 3.97	–	7.00 ± 3.61	–
	OP	5.32 ± 2.79	7.11 ± 3.87	–	7.08 ± 3.95	0.28 ± 0.89	7.00 ± 3.94
**2 overtime resources**							
Costs: baseline	IP	0.88 ± 1.10	0.06 ± 0.26	3.70 ± 3.40	–	3.74 ± 3.33	–
	OP	3.01 ± 2.49	3.56 ± 3.29	–	3.66 ± 3.32	0.02 ± 0.21	3.67 ± 3.37
Costs: low	IP	0.83 ± 1.07	0.08 ± 0.29	3.31 ± 3.24	–	3.55 ± 3.29	–
	OP	2.79 ± 2.46	3.65 ± 3.39	–	3.71 ± 3.36	0.02 ± 0.22	3.48 ± 3.27
Costs: high	IP	0.88 ± 1.11	0.02 ± 0.17	3.63 ± 3.36	–	3.79 ± 3.33	–
	OP	2.97 ± 2.48	3.50 ± 3.28	–	3.60 ± 3.33	0.02 ± 0.22	3.43 ± 3.28

**Table 7:** Number of IPs and OPs not served under each policy considering different scenarios, with IP and EP arrival probabilities at the low level

		Policies					
	Type	Optimal	P1	P2	P3	P4	P5
**Double overbooking**							
**1 overtime resource**							
Costs: baseline	IP	0.14 ± 0.42	0.02 ± 0.16	1.02 ± 1.80	–	1.03 ± 1.79	–
	OP	0.91 ± 1.53	1.01 ± 1.78	–	1.10 ± 1.89	–	0.87 ± 1.66
Costs: low	IP	0.13 ± 0.41	0.02 ± 0.21	0.95 ± 1.75	–	0.97 ± 1.75	–
	OP	0.86 ± 1.48	0.98 ± 1.72	–	0.89 ± 1.71	–	0.94 ± 1.72
Costs: high	IP	0.14 ± 0.43	0.02 ± 0.16	0.96 ± 1.76	–	0.98 ± 1.77	–
	OP	0.88 ± 1.51	0.80 ± 1.60	–	0.98 ± 1.79	–	0.91 ± 1.74
**2 overtime resources**							
Costs: baseline	IP	0.01 ± 0.12	0.00 ± 0.00	0.12 ± 0.61	–	0.13 ± 0.63	–
	OP	0.12 ± 0.53	0.13 ± 0.64	–	0.13 ± 0.64	–	0.15 ± 0.66
Costs: low	IP	0.01 ± 0.13	0.00 ± 0.00	0.12 ± 0.61	–	0.13 ± 0.62	–
	OP	0.11 ± 0.52	0.16 ± 0.69	–	0.15 ± 0.67	–	0.11 ± 0.56
Costs: high	IP	0.01 ± 0.14	0.00 ± 0.00	0.14 ± 0.66	–	0.13 ± 0.63	–
	OP	0.13 ± 0.57	0.13 ± 0.64	–	0.15 ± 0.69	–	0.12 ± 0.58
**Flight overbooking**							
**1 overtime resource**							
Costs: baseline	IP	0.11 ± 0.38	0.02 ± 0.18	0.79 ± 1.69	–	0.85 ± 1.71	–
	OP	0.65 ± 1.34	0.83 ± 1.69	–	0.67 ± 1.55	–	0.78 ± 1.65
Costs: low	IP	0.12 ± 0.40	0.01 ± 0.14	0.75 ± 1.62	–	0.83 ± 1.69	–
	OP	0.70 ± 1.39	0.67 ± 1.51	–	0.72 ± 1.57	–	0.80 ± 1.67
Costs: high	IP	0.10 ± 0.38	0.00 ± 0.00	0.73 ± 1.56	–	0.82 ± 1.69	–
	OP	0.63 ± 1.30	0.79 ± 1.63	–	0.78 ± 1.63	–	0.74 ± 1.60
**2 overtime resources**							
Costs: baseline	IP	0.01 ± 0.11	0.00 ± 0.00	0.11 ± 0.58	–	0.10 ± 0.57	–
	OP	0.08 ± 0.44	0.12 ± 0.61	–	0.10 ± 0.56	–	0.11 ± 0.59
Costs: low	IP	0.01 ± 0.13	0.00 ± 0.00	0.11 ± 0.60	–	0.12 ± 0.63	–
	OP	0.09 ± 0.48	0.09 ± 0.53	–	0.10 ± 0.57	–	0.09 ± 0.51
Costs: high	IP	0.01 ± 0.11	0.00 ± 0.00	0.12 ± 0.60	–	0.10 ± 0.55	–
	OP	0.09 ± 0.49	0.11 ± 0.57	–	0.10 ± 0.57	–	0.12 ± 0.63

**Table 8:** Number of IPs and OPs not served under each policy considering different scenarios, with IP and EP arrival probabilities at the high level

	Type	Policies					
		Optimal	P1	P2	P3	P4	P5
**Double overbooking**							
**1 overtime resource**							
Costs: baseline	IP	5.11 ± 1.69	0.43 ± 1.03	16.60 ± 3.88	–	13.75 ± 2.21	–
	OP	11.56 ± 2.62	16.04 ± 3.81	–	16.56 ± 3.84	2.57 ± 2.66	16.61 ± 3.84
Costs: low	IP	5.01 ± 1.69	0.60 ± 1.18	16.55 ± 3.89	–	13.83 ± 2.17	–
	OP	11.49 ± 2.64	15.53 ± 3.95	–	16.54 ± 3.83	2.73 ± 2.71	16.34 ± 3.86
Costs: high	IP	5.16 ± 1.70	0.24 ± 0.77	16.41 ± 3.89	–	13.81 ± 2.16	–
	OP	11.32 ± 2.62	16.25 ± 3.79	–	16.98 ± 3.80	2.66 ± 2.69	16.43 ± 3.86
**2 overtime resources**							
Costs: baseline	IP	3.71 ± 1.61	0.08 ± 0.49	12.38 ± 3.88	–	11.87 ± 3.16	–
	OP	8.67 ± 2.67	12.20 ± 3.86	–	12.26 ± 3.86	0.63 ± 1.39	12.52 ± 3.84
Costs: low	IP	3.70 ± 1.59	0.25 ± 0.74	12.99 ± 3.83	–	11.83 ± 3.14	–
	OP	8.67 ± 2.66	12.23 ± 3.83	–	12.68 ± 3.86	0.59 ± 1.32	12.35 ± 3.88
Costs: high	IP	3.88 ± 1.62	0.12 ± 0.55	12.62 ± 3.82	–	11.73 ± 3.18	–
	OP	8.73 ± 2.58	12.45 ± 3.93	–	12.80 ± 3.84	0.58 ± 1.34	12.58 ± 3.84
**Flight overbooking**							
**1 overtime resource**							
Costs: baseline	IP	5.20 ± 1.76	0.36 ± 0.95	16.65 ± 3.89	–	13.77 ± 2.21	–
	OP	11.17 ± 2.60	15.94 ± 3.87	–	16.47 ± 3.89	2.54 ± 2.64	15.96 ± 3.93
Costs: low	IP	5.22 ± 1.77	0.57 ± 1.15	16.41 ± 3.91	–	13.83 ± 2.19	–
	OP	11.32 ± 2.62	15.80 ± 3.97	–	16.26 ± 3.88	2.72 ± 2.70	16.39 ± 3.92
Costs: high	IP	5.35 ± 1.78	0.31 ± 0.93	16.26 ± 3.96	–	13.82 ± 2.16	–
	OP	11.28 ± 2.49	15.98 ± 3.99	–	16.53 ± 3.83	2.68 ± 2.69	16.45 ± 3.91
**2 overtime resources**							
Costs: baseline	IP	3.73 ± 1.68	0.14 ± 0.48	12.20 ± 3.88	–	11.91 ± 3.17	–
	OP	8.32 ± 2.62	12.27 ± 3.96	–	12.42 ± 3.91	0.69 ± 1.46	12.76 ± 3.85
Costs: low	IP	3.85 ± 1.69	0.29 ± 0.64	12.47 ± 3.94	–	11.74 ± 3.20	–
	OP	8.49 ± 2.58	12.26 ± 3.89	–	12.67 ± 3.90	0.58 ± 1.32	12.36 ± 3.85
Costs: high	IP	3.84 ± 1.69	0.06 ± 0.40	12.57 ± 3.90	–	11.86 ± 3.15	–
	OP	8.28 ± 2.54	12.33 ± 3.83	–	12.42 ± 3.91	0.65 ± 1.42	12.54 ± 3.90

Under the optimal policy, the average number of patients unserved was smaller when two resources were allocated in overtime periods. Considering the averages of the 3 costs’ levels, the average reductions were: (*i*) when ${p}_i^{\mathrm{IP}}=0.60$ and ${p}_i^{\mathrm{EP}}=0.15$, 52% reduction for IP and 46% for OP (“double” overbooking), and 51 and 46% reductions for IP and OP, respectively (“flight” overbooking) (Table 6); (*ii*) when ${p}_i^{\mathrm{IP}}=0.40$ and ${p}_i^{\mathrm{EP}}=0.10$, 93% reduction for IP and 86% for OP (“double” overbooking), and 91 and 87% reductions for IP and OP, respectively (“flight” overbooking) (Table 7); (*iii*) when ${p}_i^{\mathrm{IP}}=0.80$ and ${p}_i^{\mathrm{EP}}=0.20$, 26% reduction for IP and 24% for OP (“double” overbooking), and 28 and 25% reductions for IP and OP, respectively (“flight” overbooking) (Table 8). Reductions in the average number of unserved patients led to lower average total costs with 2 resources allocated in overtime periods, regardless of remaining configurations.

Tables 6, 7, and 8 also show that the optimal policy promotes a balance between the number of IPs and OPs not served, while remaining policies penalize only one type of patient due to their patient selection criteria.

## Discussion

Our study contributes to the literature by incorporating into the dynamic capacity allocation problem two aspects that best represent the reality of many radiology departments. First, we showed that the number of available resources has implications not only regarding the set of feasible actions in each service period but also regarding the set of possible states, demonstrated through restrictions that limit the sum of IPs and OPs waiting for service at the start of each period. Such restrictions contribute to minimizing the impact of the “dimensionality curse” present in real problems with large state spaces, which in turn limit the application of traditional dynamic programming methods in determining optimal policies of an MDP model. In the model presented here, if states were created considering only lower and upper limits of each patient type waiting for service, disregarding restrictions related to capacities in each service period, 93,534 possible states would be generated under “double” overbooking and allocation of two overtime resources. Applying the proposed restrictions, the number of states reduces by approximately 43%, to 52,680.

The second contribution is associated with the fact that OP no-show probabilities were obtained from a statistical model that considers the characteristics of patients and appointment schedules. In the MDP model proposed here, individual no-show probabilities are considered in the calculation of transition probabilities between system stages, which was not considered in the related literature. The extensive numerical analyses carried out under different scenarios based on parameters obtained in a case study allowed us to validate the optimal policy in comparison with 5 alternative policies, two of which were not covered in previous studies (P4 and P5). We reinforced conclusions from similar studies which attest to the superiority of optimal policies over intuitive policies [16, 17].

The implementation of the model proposed here can effectively improve the efficiency of the analyzed radiology department, maximizing the service rates of SUS patients. Our model may be easily adopted by managers of radiology departments to allocate available capacity. For example: consider the case where in the first period (*i* = 1) 3 OPs $$ \left({Ag}_1^{\mathrm{OP}}=3\right) $$ are scheduled under the double overbooking rule, and there are 3 resources available for service (*C*_1_ = 3). Let *z*_1_ = (1, 3, 1) be the observed system state, meaning that 1 IP, 3 OPs, and 1 EP await service. In this case, the model indicates that there are two feasible actions for this state, given the available capacity: $$ {A}_{z_1}=\left(0,2,1\right) $$ or $$ {A}_{z_1}=\left(1,1,1\right) $$. The model indicates that the best decision is the one with the lowest costs, that is, $$ {A}_{z_1}=\left(0,2,1\right) $$ that represents selecting for service 0 IPs, 2 OPs, and 1 EP at the cost of US$ 16.37. In Additional file 8, a spreadsheet is provided with the solution presented by the model for the problem considering a double overbooking rule, two resources allocated in overtime periods, cost levels and probabilities of arrival of IPs and EPs set at the base levels. By informing the period of service and the numbers of IPs, OPs, and EPs waiting for service in the spreadsheet, managers may visualize the decision with the lowest associated cost.

Following the guidelines above, it is noteworthy that the application of the model is not restricted to the Brazilian case, being easily adaptable to radiology facilities in other countries. For that, the analyst must inform the values of the model parameters that characterize the system of interest (the list of parameters is available in Table 1). Changes in parameter values tested in the numerical analysis reported here demonstrated the robustness of the optimal policy, enabling it to be implemented at any radiology facility, regardless of size and demand for exams.

Implications of using our proposed model are mainly associated with efficiency improvements in the use of CT resources, costs and/or revenues. For example, considering the recommendation to use the optimal policy, under a double overbooking rule, two resources in overtime, and IPs and EPs arrival probabilities at baseline levels, the average occupancy rate observed for the system was 97.16%. Previous studies proposing similar approaches and using real data from radiology services in other countries reported resource utilization rates close to those observed here. Patrick et al. [15] used data from a radiology service in Canada. They observed that the occupancy rate for CT resources ranged between 96.60 and 99.85% using the optimal policy, considering various combinations of scenarios. Schütz and Kolisch [17] reported an average utilization rate of 91.20% for MRI resources in a radiology department of a university hospital located in Germany.

The model proposed in this article may also be applied to other types of radiology resources, such as X-ray, ultrasound, and magnetic resonance, without demanding significant changes in its structure. Further, our model may be adapted to consider stochastic service times, although the assumption of deterministic times is aligned with similar studies focusing on Radiology services listed in Table S1 (Additional file 1).

Extensions of this research may include the design of overbooking rules that allows optimized scheduling of overtime patients. In addition, the proposed model could be extended to include other factors, such as OPs that leave the system without being served after long waits incurring a penalty cost for dropping out.

## Conclusions

The optimal policy obtained by the proposed model showed superior performance (lowest total daily cost) compared to alternative policies when considering the premises and parameters established in the present work. Guidelines for the practical implementation of this policy were provided with relative ease so that by observing the state of the system at any time (number of patients waiting for the service), managers of the radiology services can make a decision (number and types of patients which must be admitted for service) so that the cost of the system is minimized.

The alternative policy that prioritizes outpatients for care in relation to inpatients (policy P2) was the one that performed closest to the optimal policy and is also easy to implement in practice.

## Supplementary Information

**Additional file 1:.** Summary of related literature on capacity allocation in imaging facilities.

**Additional file 2:.** Predictive no-show model.

**Additional file 3:.** Deriving the sets of possible states for regular and overtime service periods.

**Additional file 4:.** Script with the programming code in SciLab.

**Additional file 5;.** Histograms of total costs of 10,000 days of service simulated with IPs and EPs probabilities at baseline level - cost levels (low and high).

**Additional file 6:.** Histograms of total costs obtained simulating 10,000 service days with IPs and EPs probabilities at the low level - cost levels (low and high).

**Additional file 7:.** Histograms of total costs obtained simulating 10,000 service days with IPs and EPs probabilities at the high level - cost levels (low and high).

**Additional file 8:.** Solution presented by the model for the problem.

## Data Availability

Based on a mutual agreement between researchers and Radiology Department of Hospital de Clínicas de Porto Alegre (HCPA), the datasets used during the present study are not publicly available.

## Abbreviations

MDP: Markov decision process
DP: Dynamic programming
CT: Computed tomography
MRI: Magnetic resonance imaging
SUS: Sistema unico de saude
