# Supplementary material for: Dynamic capacity allocation in a radiology service considering different types of patients, individual no-show probabilities, and overbooking
Source: BMC Health Serv Res. 2021 Sep 14;21:968. doi: 10.1186/s12913-021-06918-y (PMC8442351; doi:10.1186/s12913-021-06918-y)
Supplement: Supplementary file 1 — Additional file 1:. Summary of related literature on capacity allocation in imaging facilities. [file 12913_2021_6918_MOESM1_ESM.docx]

**Table S1** Summary of related literature on capacity allocation in imaging facilities

| **Authors**  **(in chronological order)** | **Objective: minimize (Min) or maximize (Max)** | **Classes of patients** | **Service time** | **Type/Number of resources** | **Arrival probabilities** |
| --- | --- | --- | --- | --- | --- |
| Green et al. [14] | Max net revenue (revenues deducted of waiting and penalty costs) | Inpatients, outpatients and emergency | Deterministic | Magnetic Resonance Imaging (MRI)/Single resource | Deterministic |
| Kolisch and Sickinger [1] | Max net revenue (revenues deducted of waiting and penalty costs) | Inpatients, outpatients and emergency | Deterministic | Computed Tomography (CT)/Multiple resources | Deterministic |
| Patrick et al. [15] | Min costs of patient  rejection, booking  patient, and waiting time | Inpatients and outpatients | Deterministic | CT/Multiple resources | Stochastic |
| Gocgun et al. [16] | Max net revenue (revenues deducted of waiting and penalty costs) | Inpatients, outpatients and emergency patients (critical and non-critical) | Deterministic | CT/Multiple resources | Stochastic |
| Schütz and Kolisch [5] | Max profit (revenue from  patients served – waiting time, overtime,  and patient rejection costs) | Inpatients and outpatients | Stochastic | MRI/Single resource | Stochastic |
| Schütz and Kolisch [17] | Max profit (revenue of  patients served – patient rejection and  overtime costs) | Inpatients and outpatients | Deterministic | MRI/Single resource | Stochastic |
| Xiao and Zhu [2] | Max revenue of patients served | Outpatients and emergency | Stochastic | MRI or CT/Single resource | Deterministic |
| Geng and Xie [18] | Max revenue of patients served | Outpatients | Deterministic | MRI or CT/Single resource | Stochastic |
| Geng et al. [4] | Min waiting and penality costs | Inpatients and outpatients | Deterministic | MRI/Single resource | - |
