# Supplementary material for: Dynamic capacity allocation in a radiology service considering different types of patients, individual no-show probabilities, and overbooking
Source: BMC Health Serv Res. 2021 Sep 14;21:968. doi: 10.1186/s12913-021-06918-y (PMC8442351; doi:10.1186/s12913-021-06918-y)
Supplement: Supplementary file 2 — Additional file 2:. Predictive no-show model. [file 12913_2021_6918_MOESM2_ESM.docx]

**Predictive no-show model**

We performed a retrospective study using outpatient computed tomography (CT) scan scheduling data obtained from the consultation scheduling management system of the Radiology Department of a public, 850-bed, tertiary care teaching hospital in southern Brazil.

We considered in the study appointment records from patients that visited the hospital at least once in 2016 for medical consultations, laboratory or radiology exams. In 2017, 12,387 appointments were made for CT exams; 2,966 did not meet our first screening selection criteria. The 9,421 remaining appointment records covered a one-year period, from Jan 1 to Dec 31, 2017. In the final sample analyzed we removed patients with more than one CT appointment on the same day, which led to 8,382 appointments (and a total of 557 no-shows). Our dataset did not include appointments with missing information on the analyzed predictors.

The dataset carries information on 15 categorical and continuous predictors (Gender, Age, Race, Marital status, Schooling, Distance, Cancer, Day, Shift, Month, Lead time, Number of consultations scheduled in previous year, Number of no-shows to consultations in the previous year, Number of exams scheduled in previous year, Number of exams scheduled in previous year, and Number of no-shows to exams in the previous year). Variables included in the dataset were chosen based on the literature and availability of data in the scheduling system. The response (dependent) variable was defined to be the outpatient attendance status with two possible outcomes: show or no-show. The primary outcome (no-show) was associated with a patient that did not show to perform the scheduled CT or did not cancel the appointment.

The dataset was split into training and test portions. Records in each portion were randomly selected, however keeping the same proportion of no-shows in the complete sample (6.65%). The training portion was comprised of 5,867 records (403 no-shows) and used to develop the models; the test portion was comprised of 2,515 records (154 no-shows) and used to validate the models. Model performance was evaluated using the Area Under the Curve (AUC) of the Receiver Operating Characteristic (ROC) for training and test portions.

Logistic regression with LASSO penalization (Least Absolute Shrinkage and Selection Operator) was used to analyze the factors associated with patient's no-show in the training portion of the dataset. Penalized logistic regression is a technique similar to logistic regression, although used for automatic selection of variables. For that, it imposes a penalty on the model *β* coefficients to induce dispersion, such that coefficients of less relevant predictors are reduced to zero [1]. This process results in biased coefficient estimates that can no longer be interpreted as odds ratios, as in a logistic regression, but rather as a weight or relative importance of the predictor variable [2].

The objective was to choose a model to calculate patients’ no-show probabilities to scheduled CT exam appointments. Using the test portion of the dataset the selected model goodness-of-fit was assessed using the Hosmer–Lemeshow test, which divides observations into classes based on their predicted no-show probabilities and calculates the chi-squared value associated with observed and expected frequencies in each class [3]. All analyses were performed using the R package (version 3.5.1).

Regression coefficients (*β*) of the penalized logistic regression model, determined using the training dataset, are shown in Table A. Predictors with positive (negative) associated regression coefficients are indicators of higher (lower) no-show probability.

The model displayed an AUC of 0.741 in the training portion and 0.774 in the testing portion. The Hosmer–Lemeshow test validated the penalized logistic regression model applied to the test portion of the dataset, i.e. the difference between the observed and predicted probabilities was not significant (*p* = 0.135).

**Table A** Regression coefficients of the penalized logistic regression model

| **Predictors** | **Type** | ***β*** |
| --- | --- | --- |
| Intercept |  | -3.10 |
| Race | Categorical |  |
| Not white |  | 0.21 |
| Marital Status | Categorical |  |
| Married |  | -0.12 |
| Divorced/Separated |  | -0.09 |
| Month | Categorical |  |
| June |  | 0.34 |
| October |  | -1.00 |
| November |  | -1.18 |
| December |  | -1.31 |
| Number of no-shows to exams in the previous year | Categorical |  |
| 1 |  | 0.17 |
| 2 |  | 0.30 |
| 3 or more |  | 0.20 |
| Log (Distance) | Continuous | -0.02 |
| Log (Lead time) | Continuous | 0.16 |
| Number of no-shows to consultations in previous year | Continuous | 0.03 |
| Log (Number of exams scheduled in previous year) | Continuous | -0.02 |

Using the penalized logistic regression model with *β* coefficients presented in Table A it is possible to determine the no-show probability of patients with scheduled CT exams. For that, we input values observed for the variables which are significant predictors (Table A), associated with a specific patient.

To illustrate, consider Eqn. (1):

|  | *P*(no-show) = $\frac{\text{e}^{\text{g}\text{(}\text{x}\text{)}}}{\text{1+ }\text{e}^{\text{g}\text{(}\text{x}\text{)}}}$ | (1) |
| --- | --- | --- |

where *P*(no-show) denotes the no-show probability, *e* denotes the exponential operator, and *g*(*x*) is the regression equation obtained with coefficients *β* of the penalized logistic regression in Table A. Eqn. (2) gives *g*(*x*) and its significant predictors:

| 𝑔(𝑥) = −3.10 + 0.21(Race = Not white) − 0.12(Marital Status = Married) − 0.09(Marital Status = Divorced/Separated) + 0.34(Month = June) – 1.00(Month = October) − 1.18(Month = November) – 1.31(Month = December) + 0.17(Number of no-shows to exams in the previous year = 1) + 0.30(Number of no-shows to exams in the previous year = 2) + 0.20(Number of no-shows to exams in the previous year = 3) − 0.02Log(Distance) + 0.16Log(Lead-time) + 0.03(Number of no-shows to consultations in previous year) − 0.02Log(Number of exams scheduled in previous year) | (2) |
| --- | --- |

**References**

1. Lin Q, Betancourt B, Goldstein BA, Steorts RC. Prediction of appointment no-shows using electronic health records. J Appl Stat. 2019; 1–15.

2. Ding X, Gellad ZF, Mather C, Barth P, Poon EG, Newman M, et al. Designing risk prediction models for ambulatory no-shows across different specialties and clinics. J Am Med Informatics Assoc. 2018; 25(8): 924–30.

3. Hosmer DW, Lemeshow S, Sturdivant RX. Applied Logistic Regression. 3rd ed. Hoboken, editor. New Jersey: John Wiley & Sons, Inc.,; 2013. 528 p.
