# Supplementary material for: Dynamic capacity allocation in a radiology service considering different types of patients, individual no-show probabilities, and overbooking
Source: BMC Health Serv Res. 2021 Sep 14;21:968. doi: 10.1186/s12913-021-06918-y (PMC8442351; doi:10.1186/s12913-021-06918-y)
Supplement: Supplementary file 3 — Additional file 3:. Deriving the sets of possible states for regular and overtime service periods. [file 12913_2021_6918_MOESM3_ESM.docx]

**Deriving the sets of possible states for regular and overtime service periods**

- **Set of possible states for regular service periods,** $\text{i}$

To obtain the set of all possible states $\text{(}\text{Z}_{\text{i}}\text{)}$ at the start of each regular service period $\text{i}$, consider the following assumptions:

(i) $\text{I}\text{Ps}$ and $\text{E}\text{Ps}$ may arrive at $\text{i}\text{ = 1}$, in opposition to the assumption in Kolisch and Sickinger [1];

(ii) Associated with each regular period $\text{i}$ there is a service capacity $\text{C}_{\text{i}}$;

(iii) An $\text{E}\text{P}$ arriving in time interval ($\text{t}\text{i}\text{-1}$, $\text{t}_{\text{i}}]$ will be served in interval ($\text{t}_{\text{i}}$, $\text{t}_{\text{i}\text{+1}}$];

(iv) At each regular period $\text{i}$, not more than 1 (one) $\text{I}\text{P}$ and $\text{E}\text{P}$ will arrive;

(v) $\text{Ag}_{\text{i}}^{\text{OP}}$ gives the number of $\text{O}\text{Ps}$ scheduled for service at period $\text{i}$.

Given these assumptions, the following applies:

For $\text{i}\text{ = 1}$: the number of $\text{I}\text{Ps}$ waiting for service has an upper bound of $\text{i}$ and a lower bound of 0; the number of $\text{O}\text{Ps}$ waiting for service has an upper bound of $\text{Ag}_{\text{i}}^{\text{OP}}$and a lower bound of 0; the number of $\text{E}\text{Ps}$ waiting for service has an upper bound of 1 and a lower bound of 0. Thus, the set $\text{Z}_{\text{i}}$ of all possible states at the start of period $\text{i}\text{ = 1}$ is given by Eqn. (1).

For $\text{i}\text{ ≥ 2}$: at $\text{t}_{\text{i}}$ (i.e. the start of period $\text{i}$), the number of $\text{I}\text{Ps}$ waiting for service is upper bounded by $\text{i}$ (considering that only one $\text{I}\text{P}$ can arrive at any given period), i.e.$\text{ 0 ≤} \text{w}_{\text{i}}^{\text{IP}} \text{≤} \text{i}$; the number of $\text{O}\text{Ps}$ waiting for service is upper bounded by ($\text{Ag}_{\text{1}}^{\text{OP}}$+...+ $\text{Ag}_{\text{i}}^{\text{OP}}$), i.e. $\text{0 ≤}\text{ w}_{\text{i}}^{\text{OP}}\text{ ≤ }\sum_{\text{l} =1}^{\text{i}} \text{ Ag}_{\text{l}}^{\text{OP}}$; the number of $\text{E}\text{Ps}$ waiting for service is upper bounded by 1, i.e. $\text{0 ≤ }\text{ w}_{\text{i}}^{\text{EP}}\text{ ≤ 1}$.

In addition, at regular periods $\text{i}$, some $\text{I}\text{Ps}$ and/or $\text{O}\text{Ps}$ may be selected for service (even if one $\text{E}\text{P}$ arrives for service) using the remaining capacity, since ${\text{2 ≤ }\text{C}}_{\text{i}}\leq\text{3}$. Thus, to obtain the set of possible states for periods $\text{i}\text{ ≥ 2}$, a quantity $\sum_{\text{l}\text{ = 1}}^{\text{i}\text{ – 1}} \text{(}\text{C}_{\text{l}}-\text{w}_{\text{l}}^{\text{EP}}\text{)}$ must be subtracted from the number of $\text{O}\text{Ps}$ ($\sum_{\text{l} =\text{1}}^{\text{i}} \text{Ag}_{\text{l}}^{\text{OP}}\text{)}$and $\text{I}\text{Ps}$ $\text{(}\text{i}\text{)}$ that arrive for service, corresponding to the number of $\text{O}\text{Ps}$ and $\text{I}\text{Ps}$ served in previous periods, i.e. $\text{w}_{\text{i}}^{\text{IP}}\text{ + }\text{w}_{\text{i}}^{\text{OP}} \text{≤} \sum_{\text{l} =\text{1}}^{\text{i}} \text{ Ag}_{\text{l}}^{\text{OP}}+\text{i}- \sum_{\text{l}\text{ = 1}}^{\text{i}\text{ – 1}} \text{(}\text{C}_{\text{l}}-\text{w}_{\text{l}}^{\text{EP}}\text{)}$. However, the set of possible states for $\text{i}\text{ ≥ 2}$ will only use the expressions given above if the remaining capacitity from previous periods is not sufficient to serve all $\text{I}\text{Ps}$ and $\text{O}\text{Ps}$ that arrive for service, i.e. if $\sum_{\text{l} =\text{1}}^{\text{i}} \text{ Ag}_{\text{l}}^{\text{OP}}+\text{i}\geq\left[ \sum_{\text{l}\text{ = 1}}^{\text{i}\text{ - 1}} \text{(}\text{C}_{\text{l}}-\text{w}_{\text{l}}^{\text{EP}}\text{)} \right]$. Otherwise, the set of states at time $\text{t}_{\text{i}}$ will be based on the new arrivals of $\text{I}\text{Ps}$, $\text{O}\text{Ps}$ and $\text{E}\text{Ps}$, such that $\text{I}\text{Ps}$ and $\text{E}\text{Ps}$ are upper bounded by 1, and the number of $\text{O}\text{Ps}$ waiting for service is upper bounded by $\text{Ag}_{\text{i}}^{\text{OP}}$.

Considering the conditions above, the set of all possible states ${\text{(}\text{Z}}_{\text{i}}\text{)}$ at the start of regular period $\text{i}$ for $\text{i}\text{ ≥ 2}$ will be given by Eqn. (2).

- **Set of possible states for overtime service periods,** $\text{k}$

In overtime periods $\text{N}\text{+}\text{k}$ ($\text{k}\text{ = 1, …, }\text{K}\text{)}$ there will be no $\text{I}\text{P}$ or $\text{O}\text{P}$ arrivals. All $\text{E}\text{Ps}$ that arrive will be served and those arriving at $\text{N}\text{+1}$ will be served by a dedicated CT resource.

Given these assumptions, the following applies:

For $\text{k}\text{ = 1}$: at time $\text{t}_{\text{(}\text{N}\text{+}\text{k}\text{)}}$_,_ the number of $\text{I}\text{Ps}$ waiting for service is upper bounded by $\text{N}$ and the number of $\text{O}\text{Ps}$ is upper bounded by $\sum_{\text{l} =\text{1}}^{\text{N}} \text{ Ag}_{\text{l}}^{\text{OP}}$. The same condition verified for $\text{I}\text{Ps}$ and $\text{O}\text{Ps}$ waiting for service at regular periods applies here, i.e. $\text{w}_{\text{k}}^{\text{IP}}+\text{ w}_{\text{k}}^{\text{OP}}\text{ ≤ }\sum_{\text{l} =\text{1}}^{\text{N}} \text{Ag}_{\text{l}}^{\text{OP}}+\text{N -}\text{ }\left[ \sum_{\text{l}\text{ = 1}}^{\text{N}} \text{(}\text{C}_{\text{l}}\text{- }\text{w}_{\text{l}}^{\text{EP}}\text{)} \right]$.

As for regular service periods, the set of all possible states will be derived based on the above expressions if the following condition applies: $\sum_{\text{l} =\text{1}}^{\text{N}} \text{Ag}_{\text{l}}^{\text{OP}}+\text{N ≥}\left[ \sum_{\text{l}\text{ = 1}}^{\text{N}} \text{(}\text{C}_{\text{l}}\text{- }\text{w}_{\text{l}}^{\text{EP}}\text{)} \right]$; otherwise, all $\text{O}\text{Ps}$ and $\text{I}\text{Ps}$ will have been served until the last regular period $\text{N}$, and there is no need for overtime periods. In that case, the set of all possible states at $\text{t}_{\text{(}\text{N}\text{+}\text{k}\text{)}}$ corresponds to a terminal state in which: $\text{S}_{\text{k}}=\left. \begin{aligned} \begin{matrix} \left\{ \text{(}\text{w}_{\text{(}\text{N}\text{+}\text{k}\text{)}}^{\text{IP}}\text{,}\text{ }\text{w}_{\text{(}\text{N}\text{+}\text{k}\text{)}}^{\text{OP}}\text{)} | \begin{matrix} \text{w}_{\text{(}\text{N}\text{+}\text{k}\text{)}}^{\text{IP}}\text{ = 0} \\ \text{w}_{\text{(}\text{N}\text{+}\text{k}\text{)}}^{\text{OP}}\text{ = }\text{0} \end{matrix} \right\} \\ \end{matrix} \end{aligned} \right.$. Thus, the set of all possible states at the start of period $\text{k}\text{ = 1}$ will be given by Eqn. (3).

For $\text{k}\text{ ≥ 2}$: in case period $\text{N}\text{+1}$ is not a terminal state, the same constraints verified in $\text{k}\text{ = 1}$ for the number of $\text{I}\text{Ps}$ and $\text{O}\text{Ps}$ waiting for service will apply in $\text{t}_{\text{(}\text{N}\text{+}\text{k}\text{)}}$ ($\text{k}\text{ = 2, …, }\text{K}\text{)}$, except for the constraint that adds the number of $\text{I}\text{Ps}$ ($\text{w}_{\text{(}\text{N}\text{+}\text{k}\text{)}}^{\text{IP}}$) and $\text{O}\text{Ps}$ ($\text{w}_{\text{(}\text{N}\text{+}\text{k}\text{)}}^{\text{OP}}$), which will be given by $\text{w}_{\text{k}}^{\text{IP}}+\text{ w}_{\text{k}}^{\text{OP}}\text{ ≤ }\sum_{\text{l} =\text{1}}^{\text{N}} \text{Ag}_{\text{l}}^{\text{OP}}+\text{N -}\text{ }\left[ \sum_{\text{l}\text{ = 1}}^{\text{N}} \text{(}\text{C}_{\text{l}}\text{- }\text{w}_{\text{l}}^{\text{EP}}\text{) -}\sum_{\text{y}\text{ = 1}}^{\text{N}\text{ + }\text{k}\text{ - 1}} \text{C}_{\text{N}\text{ + }\text{y}} \right]$. That derives from the fact that at $\text{N}\text{+}\text{k}$, the number of $\text{I}\text{Ps}$ and/or $\text{O}\text{Ps}$ selected for service will be a function of the available capacity $\text{C}_{\text{(}\text{N}\text{+}\text{k}\text{)}}$. Thus, the set $\text{S}_{\text{k}}$ of all possible states at the start of period $\text{k}$ ($\text{k}\text{ = 2, …, }\text{K}\text{)}$ will be given by Eqn. (4).
