# Supplementary material for: Dynamic capacity allocation in a radiology service considering different types of patients, individual no-show probabilities, and overbooking
Source: BMC Health Serv Res. 2021 Sep 14;21:968. doi: 10.1186/s12913-021-06918-y (PMC8442351; doi:10.1186/s12913-021-06918-y)
Supplement: Supplementary file 4 — Additional file 4:. Script with the programming code in SciLab. [file 12913_2021_6918_MOESM4_ESM.docx]

**Programming code**

clear

//Overbooking rules

matriz_agendas=[

3 2 2 2 2 2 3 2 2 2 2 2 3 2 2 2 2 2 3 2 2 2 2 2 3 2 2 2 2 2 3 2 2 2 2 2 2;

8 2 2 2 2 2 2 2 2 2 2 2 2 2 2 2 2 2 2 2 2 2 2 2 2 2 2 2 2 2 2 2 2 2 2 2 2];

//Policies

matriz_prioridades=[

0 0 0 0 0 0 0 0 0 0 0 0 0 0 0 0 0 0 0 0 0 0 0 0 0 0 0 0 0 0 0 0 0 0 0 0 0 0 0 0 0; //P2

1 1 1 1 1 1 1 1 1 1 1 1 1 1 1 1 1 1 1 1 1 1 1 1 1 1 1 1 1 1 1 1 1 1 1 1 1 1 1 1 1; //P3

1 1 1 1 1 1 1 1 1 1 1 1 1 1 1 1 1 1 1 0 0 0 0 0 0 0 0 0 0 0 0 0 0 0 0 0 0 0 0 0 0; //P4

0 0 0 0 0 0 0 0 0 0 0 0 0 0 0 0 0 0 0 1 1 1 1 1 1 1 1 1 1 1 1 1 1 1 1 1 1 1 1 1 1]; //P5

//Service capacity in each service period (N = 37)/overtime resources (K = 4)

matriz_equipamentos=[3 2 3 2 3 2 3 2 3 2 3 2 3 2 3 2 3 2 3 2 3 2 3 2 3 2 3 2 3 2 3 2 3 2 3 2 3 1 1 1 1;

3 2 3 2 3 2 3 2 3 2 3 2 3 2 3 2 3 2 3 2 3 2 3 2 3 2 3 2 3 2 3 2 3 2 3 2 3 2 2 2 2];

//Costs

wn = 0.78; //individual waiting cost for IP

ws = 1.56; //individual waiting cost for OP

pin = 24.96; //individual penalty cost for IP

pis = 12.48; //individual penalty cost for OP

wnx = 2.76; //individual overtime cost for IP

wsx = 2.76; //individual overtime cost for OP

paramns_base = [wn ws pin pis wnx wsx];

//Arrival probability [IP OP EP]

matriz_pr=[0.60 NaN 0.15;

0.80 NaN 0.20;

0.40 NaN 0.10];

//Number of simulations

numero_simulacoes=10000;

//Scenarios

[qtd_tipo_pr,~]=size(matriz_pr);

[qtd_tipo_equi_extra,~]=size(matriz_equipamentos);

[qtd_over,periodos_servico]=size(matriz_agendas);

k=1;

for politica=1:6

for parametros=1:3

for overbook=1:qtd_over

for tipo_equi=1:qtd_tipo_equi_extra

for tipo_pr=1:qtd_tipo_pr

[prioridades,aleatorio,otimo]=parametros_politicas(politica,matriz_prioridades);

paramns=parametros_custos(parametros,paramns_base);

cenarios{k,1}=[politica parametros overbook tipo_equi tipo_pr];

cenarios{k,2}=prioridades;

cenarios{k,3}=aleatorio;

cenarios{k,4}=otimo;

cenarios{k,5}=paramns;

cenarios{k,6}=matriz_agendas(overbook,:);

cenarios{k,7}=matriz_equipamentos(tipo_equi,:);

cenarios{k,8}=matriz_pr(tipo_pr,:);

k=k+1;

end

end

end

end

end

//Arrival probability OP

MM = csvread('probs.csv');

total_simulacoes=6*3*qtd_over*qtd_tipo_equi_extra*qtd_tipo_pr;

resultado_cenario=cell(total_simulacoes,1);

disp('comecou')

for c=1:total_simulacoes

disp(c)

resultado_cenario{c}=funcao_geral(cenarios{c,2},cenarios{c,3},cenarios{c,4},cenarios{c,5},...

cenarios{c,6},cenarios{c,7},cenarios{c,8},MM,cenarios{c,1},numero_simulacoes);

end

function [prioridades,aleatorio,otimo] = parametros_politicas(cod_politica,matriz_prioridades)

// 1 - Optimal

// 2 – P1

// 3 – P2

// 4 – P3

// 5 – P4

// 6 – P5

if cod_politica==1

otimo=1;

aleatorio=0;

prioridades=matriz_prioridades(1,:); //qualquer,nao é usado

elseif cod_politica==2

otimo=0;

aleatorio=1;

prioridades=matriz_prioridades(1,:); //qualquer,nao é usado

elseif cod_politica==3

otimo=0;

aleatorio=0;

prioridades=matriz_prioridades(1,:);

elseif cod_politica==4

otimo=0;

aleatorio=0;

prioridades=matriz_prioridades(2,:);

elseif cod_politica==5

otimo=0;

aleatorio=0;

prioridades=matriz_prioridades(3,:);

elseif cod_politica==6

otimo=0;

aleatorio=0;

prioridades=matriz_prioridades(4,:);

end

end

function [paramns] = parametros_custos(cod_custos,paramns_base)

// 1 - parametro base

// 2 - 50// do parametro base

// 3 - 150// do parametro base

if cod_custos==1

paramns=paramns_base;

elseif cod_custos==2

paramns=(0.5)*paramns_base;

elseif cod_custos==3

paramns=(1.5)*paramns_base;

end

end

function [saida] = funcao_geral(prioridades,aleatorio,otimo,paramns,age,equipamentos,pr,MM,codigos,nsim)

prob_agendados=[];

for xx=1:length(age)

for yy=1:age(xx)

prob_agendados(yy,xx) = MM(randi([1 length(MM)],1,1));

end

end

Obj = progdin(age,pr,paramns,equipamentos,prob_agendados,prioridades,aleatorio,otimo);

T1 = tabela(Obj);

[~,N]=size(age);

lnteqp = length(equipamentos);

somaeq = sum(equipamentos);

somaeqE = sum(equipamentos(N+1:lnteqp));

[T,TS,HIS] = simul(paramns,Obj,age,prob_agendados,pr,N,lnteqp,somaeq,somaeqE,nsim);

function Obj = progdin(age,pr,params,equipamentos,AG,prioridades,aleatorio,otimo)

//////////STATE SPACE/ACTIONS//////////

Z=ESTADOS_ACOES(age,equipamentos,prioridades,aleatorio,otimo);

//////////DYNAMIC PROGRAMMING//////////

[~,N]=size(age);

lnteqp = length(equipamentos);

RR = cell(lnteqp,1);

for ii = lnteqp:-1:1

[m , ~] = size(Z{ii,1});

OO = [];

for i = 1:m

[mm , ~] = size(Z{ii,2}{i,1});

rr = [];

for j = 1:mm

vta = Z{ii,1}(i,:)-Z{ii,2}{i,1}(j,:);

if ii==lnteqp

bst = 0;

elseif ii>=N

bst = probs(vta,Z{ii+1,1}(:,:),[],pr,RR{ii+1,1}(:,:),[],N,ii);

else

bst = probs(vta,Z{ii+1,1}(:,:),age(ii+1),pr,RR{ii+1,1}(:,:),AG(1:age(ii+1),ii+1),N,ii);

end

rr(j) = custo(Z{ii,1}(i,:),Z{ii,2}{i,1}(j,:),params,N,lnteqp,ii)+bst;

end

[V, I] = min(rr);

OO(i,:) = [V I];

end

RR{ii,1} = OO;

end

Obj.estados = Z;

Obj.solucao = RR;

end

function Z=ESTADOS_ACOES(age,equipamentos,prioridades,aleatorio,otimo)

[~,N]=size(age);

Z=cell(N+length(equipamentos)-length(age),1);

z_aux=[];

for n=0:1

for s=0:age(1)

for e=0:1

z_aux=[z_aux;[n s e]];

acoes=estado_acoes([n s e],equipamentos(1),prioridades(1),aleatorio,otimo);

[aa,~]=size(z_aux);

Z{1,2}{aa,1}=acoes;

end

end

end

Z{1,1}=z_aux;

z_aux=[];

if sum(age(1:2))+2-(equipamentos(1:2-1)-1)>=0

for n=0:2

for s=0:sum(age(1:2))

for e=0:1

z=[n s e];

if z(1)+z(2)<=sum(age(1:2))+2-(equipamentos(1:2-1)-1)

z_aux=[z_aux;z];

acoes=estado_acoes(z,equipamentos(2),prioridades(2),aleatorio,otimo);

[aa,~]=size(z_aux);

Z{2,2}{aa,1}=acoes;

end

end

end

end

else

for n=0:1

for s=0:age(2)

for e=0:1

z=[n s e];

z_aux=[z_aux;z];

acoes=estado_acoes(z,equipamentos(2),prioridades(2),aleatorio,otimo);

[aa,~]=size(z_aux);

Z{2,2}{aa,1}=acoes;

end

end

end

end

Z{2,1}=z_aux;

for i=3:N

z_aux=[];

Z{i,2}=cell(1,1);

if sum(age(1:i))+i-sum(equipamentos(1:i-1)-ones(1,i-1))>=0

for n=0:i

for s=0:sum(age(1:i))

for e=0:1

z=[n s e];

if z(1)+z(2)<=sum(age(1:i))+i-sum(equipamentos(1:i-1)-ones(1,i-1))

z_aux=[z_aux;z];

acoes=estado_acoes(z,equipamentos(i),prioridades(i),aleatorio,otimo);

[aa,~]=size(z_aux);

Z{i,2}{aa,1}=acoes;

end

end

end

end

else

for n=0:1

for s=0:age(i)

for e=0:1

z=[n s e];

z_aux=[z_aux;z];

acoes=estado_acoes(z,equipamentos(i),prioridades(i),aleatorio,otimo);

[aa,~]=size(z_aux);

Z{i,2}{aa,1}=acoes;

end

end

end

end

Z{i,1}=z_aux;

end

for k=1:length(equipamentos)-length(age)

z_aux=[];

Z{N+k,2}=cell(1,1);

if sum(age(1:N))+N-(sum(equipamentos(1:N+k-1))-N)>=0

for n=0:N

for s=0:sum(age(1:N))

for e=0:0

z=[n s e];

if z(1)+z(2)<=sum(age(1:N))+N-(sum(equipamentos(1:N+k-1))-N)//ones(1,N+k-1))

z_aux=[z_aux;z];

acoes=estado_acoes(z,equipamentos(N+k),prioridades(N+k),aleatorio,otimo);

[aa,~]=size(z_aux);

Z{N+k,2}{aa,1}=acoes;

end

end

end

end

else

for n=0:0

for s=0:0

for e=0:0

z=[n s e];

z_aux=[z_aux;z];

acoes=estado_acoes(z,equipamentos(N+k),prioridades(N+k),aleatorio,otimo);

[aa,~]=size(z_aux);

Z{N+k,2}{aa,1}=acoes;

end

end

end

end

Z{N+k,1}=z_aux;

end

end

function acoes_politicas=estado_acoes(zz,qtd_equip,prioridade,aleatorio,otimo)

acoes=[];

for n=0:zz(1)

for s=0:zz(2)

if n+s+zz(3)==min(qtd_equip,sum(zz))

acao=[n s zz(3)];

acoes=[acoes;acao];

end

end

end

[linhas,~]=size(acoes);

if aleatorio==0 && otimo==0

//acoes=sortrows(eita,-1);

[~,bb]=min(abs(((1/(linhas-1))*(0:linhas-1))-prioridade*ones(1,linhas)));

acoes_politicas=acoes(bb,:);

elseif aleatorio==1 && otimo==0

valor=randperm(linhas);

acoes_politicas=acoes(valor(1),:);

elseif aleatorio==0 && otimo==1

acoes_politicas=acoes;

elseif aleatorio==1 && otimo==1

acoes_politicas=[];

end

end

//////////TRANSITION PROBABILITIES - IP AND EP//////////

function bst = probs(vta,V,ag,pr,RR,AG,N,p)

bst = 0;

vpr = probdst(AG);

if p<N

for j = 0:ag

vta1 = vta +[0 j 0];

pr1 = vpr(j+1)*(1-pr(1))*(1-pr(3));

a = find(sum(vta1 == V,2)==3);

bst = bst + pr1*RR(a,1);

vta2 = vta +[1 j 1];

pr2 = vpr(j+1)*pr(1)*pr(3);

a = find(sum(vta2 == V,2)==3);

bst = bst + pr2*RR(a,1);

vta3 = vta +[0 j 1];

pr3 = vpr(j+1)*(1-pr(1))*pr(3);

a = find(sum(vta3 == V,2)==3);

bst = bst + pr3*RR(a,1);

vta4 = vta +[1 j 0];

pr4 = vpr(j+1)*pr(1)*(1-pr(3));

a = find(sum(vta4 == V,2)==3);

bst = bst + pr4*RR(a,1);

end

else

a = find(sum(vta == V,2)==3);

bst = RR(a,1);

end

end

//////////COST FUNCTIONS//////////

function res = custo(z,a,params,N,periodos_equipamentos,p)

wn = params(1);

ws = params(2);

pin = params(3);

pis = params(4);

wnx = params(5);

wsx = params(6);

if N~=periodos_equipamentos

if p<N

res = (z(1)-a(1))*wn+(z(2)-a(2))*ws;

elseif p==N

res = (z(1)-a(1))*wn+(z(2)-a(2))*ws;

elseif p==periodos_equipamentos

res = (z(1)-a(1))*pin+(z(2)-a(2))*pis;

else

res = a(1)*(p-N)*wnx+a(2)*(p-N)*wsx;

end

else

res = (z(1)-a(1))*pin+(z(2)-a(2))*pis;

end

end

//////////TRANSITION PROBABILITIES - OP//////////

function P = probdst(V)

//Entrada V: vetor de probabilidades

//Saída P: vetor com as probabildades de chegar 0,1,2,...,k pacientes

k=length(V);

K=[];

N=2^(k)-1;

for i=1:N

a=dec2bin(i);

[~,n]=size(a);

A=[];

for j=1:n

A(j)=str2num(a(j));

end

K(i,k-n+1:k)=A;

end

K=[zeros(1,k);K];

[m,~]=size(K);

KK=[];

for ii=1:m

KK(ii)=sum(K(ii,:));

end

B=cell(k,1);

for u=0:k

b=find(KK==u);

B{u+1,1}=b;//K(b,:);

end

clear i j m n

for ii=1:length(B)

Q=B{ii};

S=0;

for i=1:length(Q)//para i que vai de 1 até 41 (comprimento do vetor de equipamentos)

PP=1;

alfa=K(Q(i),:);

for j=1:k

PP=PP*(V(j)^(alfa(j)))*((1-V(j))^(1-alfa(j)));

end

S=S+PP;

end

P(ii)=S;

end

P=P';

End

//////////ARRIVAL SIMULATION//////////

function [T,TS,HIS] = simul(params,Obj,age,prob_agendados,pr,N,periodos_equipamentos,somaeq,somaeqE,nsim)

for j = 1:nsim

cs = sum(rand(age(1),1)<prob_agendados(1:age(1),1));

cn = sum(rand(1,1)<pr(1));

ce = sum(rand(1,1)<pr(3));

ESN = [cn cs ce];

CH(1,:) = [cn cs ce];

SOL = Obj.estados{1,1};

ind = find(sum(SOL==ESN,2)==3);

indac = Obj.solucao{1,1}(ind,2);

act = Obj.estados{1,2}(ind);

act = act{1,1}(indac,:);

ES(1,:) = ESN;

AC(1,:) = act;

R(1) = Obj.solucao{1,1}(ind,1);

R2(1) = custo(ESN,act,params,N,periodos_equipamentos,1);

SA(1) = sum(act);

for i = 2:length(age)

ESV = ESN-act;

ESP(i,:) = ESV;

cs = sum(rand(age(i),1)<prob_agendados(1:age(i),i));

cn = sum(rand(1,1)<pr(1));

ce = sum(rand(1,1)<pr(3));

CH(i,:) = [cn cs ce];

ESN = ESV+[cn cs ce];

ES(i,:) = ESN;

SOL = Obj.estados{i,1};

ind = find(sum(SOL==ESN,2)==3);

indac = Obj.solucao{i,1}(ind,2);

act = Obj.estados{i,2}(ind);

act = act{1,1}(indac,:);

AC(i,:) = act;

SA(i) = sum(act);

R(i) = Obj.solucao{i,1}(ind,1);

R2(i) = custo(ESN,act,params,N,periodos_equipamentos,i);

end

for i = N+1:periodos_equipamentos

ESV = ESN-act;

ESP(i,:) = ESV;

cs = 0;

cn = 0;

ce = 0;

CH(i,:) = [cn cs ce];

ESN = ESV+[cn cs ce];

ES(i,:) = ESN;

SOL = Obj.estados{i,1};

ind = find(sum(SOL==ESN,2)==3);

indac = Obj.solucao{i,1}(ind,2);

act = Obj.estados{i,2}(ind);

act = act{1,1}(indac,:);

AC(i,:) = act;

SA(i) = sum(act);

R(i) = Obj.solucao{i,1}(ind,1);

R2(i) = custo(ESN,act,params,N,periodos_equipamentos,i);

end

RM(j) = R(end);

RS(j) = sum(R2);

OC(j) = sum(SA(1:N))/(somaeq-somaeqE);

OCE(j) = sum(SA(N+1:end))/somaeqE;

SFn(j) = ESN(1)-act(1);

SFs(j) = ESN(2)-act(2);

end

CustoPol = [mean(RS);std(RS);RS'];

CustoOtm = [mean(RM);std(RM);RM'];

Ocupacao = [mean(OC);std(OC);OC'];

OcupacaoE = [mean(OCE);std(OCE);OCE'];

SobraN = [mean(SFn);std(SFn);SFn'];

SobraS = [mean(SFs);std(SFs);SFs'];

TS = table(CustoPol,CustoOtm,Ocupacao,OcupacaoE,SobraN,SobraS);

T = table(CH,ES,AC,ESP);

//figure(1)

HIS.RS = histogram(RS);

//figure(2)

HIS.RM = histogram(RM);

//figure(3)

HIS.OC = histogram(OC);

//figure(4)

HIS.OCE = histogram(OCE);

//figure(5)

HIS.SFn = histogram(SFn);

//figure(6)

HIS.SFs = histogram(SFs);

end

function T = tabela(Obj)

n = []; s = []; e = []; an = []; as = []; ae = []; Pe = []; R = [];

for i = 1:length(Obj.solucao)

for j = 1:size(Obj.solucao{i,1},1)

Pe = [Pe;i];

n = [n; Obj.estados{i,1}(j,1)];

s = [s; Obj.estados{i,1}(j,2)];

e = [e; Obj.estados{i,1}(j,3)];

act = Obj.solucao{i}(j,2);

an = [an; Obj.estados{i,2}{j}(act,1)];

as = [as; Obj.estados{i,2}{j}(act,2)];

ae = [ae; Obj.estados{i,2}{j}(act,3)];

R = [R;Obj.solucao{i}(j,1)];

end

end

T = table(Pe,n,s,e,an,as,ae,R);

// writetable(T,'tabela.xlsx')

end

saida{1,1}=codigos;

saida{1,2}=T;

saida{1,3}=TS;

saida{1,4}=T1;

saida{1,5}=HIS;

end
