# Supplementary material for: Dynamic capacity allocation in a radiology service considering different types of patients, individual no-show probabilities, and overbooking
Source: BMC Health Serv Res. 2021 Sep 14;21:968. doi: 10.1186/s12913-021-06918-y (PMC8442351; doi:10.1186/s12913-021-06918-y)
Supplement: Supplementary file 7 — Additional file 7:. Histograms of total costs obtained simulating 10,000 service days with IPs and EPs probabilities at the high level - cost levels (low and high). [file 12913_2021_6918_MOESM7_ESM.docx]

**Figure S7.1:** (A) Histograms of total costs obtained simulating 10.000 service days with double overbooking, 1 (one) resource in overtime and costs at the low level ($\text{I}\text{Ps}$ and $\text{E}\text{Ps}$ probabilities at the high level); (B) Histograms of total costs obtained simulating 10.000 service days with double overbooking, 2 (two) resources in overtime and costs at the low level ($\text{I}\text{Ps}$ and $\text{E}\text{Ps}$ probabilities at the high level)

**Figura S7.2:** (A) Histograms of total costs obtained simulating 10.000 service days with double overbooking, 1 (one) resource in overtime and costs at the high level ($\text{I}\text{Ps}$ and $\text{E}\text{Ps}$ probabilities at the high level); (B) Histograms of total costs obtained simulating 10.000 service days with double overbooking, 2 (two) resources in overtime and costs at the high level ($\text{I}\text{Ps}$ and $\text{E}\text{Ps}$ probabilities at the high level)

**Figura S7.3:** (A) Histograms of total costs of 10,000 days of service simulated with flight overbooking, 1 (one) overtime resource and costs at low level ($\text{I}\text{Ps}$ and $\text{E}\text{Ps}$ probabilities at the high level); (B) Histograms of total costs of 10,000 days of service simulated with "flight" overbooking, 2 (two) overtime resources and costs at low level ($\text{I}\text{Ps}$ and $\text{E}\text{Ps}$ probabilities at the high level)

**Figura S7.4:** (A) Histograms of total costs of 10,000 days of service simulated with flight overbooking, 1 (one) overtime resource and costs at high level ($\text{I}\text{Ps}$ and $\text{E}\text{Ps}$ probabilities at the high level; (B) Histograms of total costs of 10,000 days of service simulated with "flight" overbooking, 2 (two) overtime resources and costs at high level ($\text{I}\text{Ps}$ and $\text{E}\text{Ps}$ probabilities at the high level)
